# Memantine treatment does not affect compulsive behavior or frontostriatal connectivity in an adolescent rat model for quinpirole-induced compulsive checking behavior

**DOI:** 10.1007/s00213-022-06139-z

**Published:** 2022-04-14

**Authors:** Milou Straathof, Erwin L. A. Blezer, Christel E. Smeele, Caroline van Heijningen, Annette van der Toorn, Jan K. Buitelaar, Jeffrey C. Glennon, Willem M. Otte, Rick M. Dijkhuizen, TACTICS Consortium, Jan Buitelaar, Saskia de Ruiter, Jilly Naaijen, Sophie Akkermans, Maarten Mennes, Marcel Zwiers, Shahrzad Ilbegi, Leonie Hennissen, Jeffrey Glennon, Ilse van de Vondervoort, Katarzyna Kapusta, Natalia Bielczyk, Houshang Amiri, Martha Havenith, Barbara Franke, Geert Poelmans, Janita Bralten, Tom Heskes, Elena Sokolova, Perry Groot, Steven Williams, Declan Murphy, David Lythgoe, Muriel Bruchhage, Iulia Dud, Bogdan Voinescu, Ralf Dittmann, Tobias Banaschewski, Daniel Brandeis, Konstantin Mechler, Ruth Berg, Isabella Wolf, Alexander Häge, Michael Landauer, Sarah Hohmann, Regina Boecker-Schlier, Matthias Ruff, Rick Dijkhuizen, Erwin Blezer, Milou Straathof, Kajo van der Marel, Pim Pullens, Wouter Mol, Annette van der Toorn, Willem Otte, Caroline van Heijningen, Sarah Durston, Vincent Mensen, Bob Oranje, René Mandl, Daphna Joel, John Cryan, Tracey Petryshen, David Pauls, Mai Saito, Angelique Heckman, Sabine Bahn, Ameli Schwalber, Ioana Florea

**Affiliations:** 1 Biomedical MR Imaging and Spectroscopy Group, Center for Image Sciences, University Medical Center Utrecht & Utrecht University, Heidelberglaan 100, 3584 CX Utrecht, the Netherlands; 2 Department of Cognitive Neuroscience, Donders Institute for Brain, Cognition and Behavior, Radboud University Medical Center, Nijmegen, the Netherlands; 3 Karakter Child and Adolescent Psychiatry University Center, Nijmegen, the Netherlands; 4 Conway Institute of Biomolecular and Biomedical Research, School of Medicine, University College Dublin, Dublin 4, Ireland; 5 Department of Pediatric Neurology, UMC Utrecht Brain Center, University Medical Center Utrecht and Utrecht University, Utrecht, the Netherlands

**Keywords:** Compulsive behavior, NMDA antagonist, Functional magnetic resonance imaging, Diffusion magnetic resonance imaging, Frontostriatal circuitry

## Abstract

**Rationale:**

Compulsivity often develops during childhood and is associated with elevated glutamate levels within the frontostriatal system. This suggests that anti-glutamatergic drugs, like memantine, may be an effective treatment.

**Objective:**

Our goal was to characterize the acute and chronic effect of memantine treatment on compulsive behavior and frontostriatal network structure and function in an adolescent rat model of compulsivity.

**Methods:**

Juvenile Sprague–Dawley rats received repeated quinpirole, resulting in compulsive checking behavior (*n* = 32; compulsive) or saline injections (*n* = 32; control). Eight compulsive and control rats received chronic memantine treatment, and eight compulsive and control rats received saline treatment for seven consecutive days between the 10th and 12th quinpirole/saline injection. Compulsive checking behavior was assessed, and structural and functional brain connectivity was measured with diffusion MRI and resting-state fMRI before and after treatment. The other rats received an acute single memantine (compulsive: *n* = 12; control: *n* = 12) or saline injection (compulsive: *n* = 4; control: *n* = 4) during pharmacological MRI after the 12th quinpirole/saline injection. An additional group of rats received a single memantine injection after a single quinpirole injection (*n* = 8).

**Results:**

Memantine treatment did not affect compulsive checking nor frontostriatal structural and functional connectivity in the quinpirole-induced adolescent rat model. While memantine activated the frontal cortex in control rats, no significant activation responses were measured after single or repeated quinpirole injections.

**Conclusions:**

The lack of a memantine treatment effect in quinpirole-induced compulsive adolescent rats may be partly explained by the interaction between glutamatergic and dopaminergic receptors in the brain, which can be evaluated with functional MRI.

**Supplementary Information:**

The online version contains supplementary material available at 10.1007/s00213-022-06139-z.

## Introduction

Compulsivity is the repetitive, irresistible urge to perform certain behaviors without voluntary control. It can be considered to be a cross-disorder trait of psychiatric disorders like obsessive–compulsive disorder (OCD) and autism spectrum disorder (ASD) (Jacob et al. 2009). Current treatment strategies for these disorders typically focus on diminishing symptoms associated with a specific diagnosis. For people with OCD, medication treatments with selective serotonin reuptake inhibitors (SSRIs) or combination treatments with cognitive-behavioral therapy have been proven effective, but 40–60% of these patients have treatment-resistant symptoms (Franklin and Foa 2011). There are no curative or even symptomatic treatments available for the key symptoms of ASD (Accordino et al. 2016), and SSRI treatment is not very effective (Williams et al. 2013). This stresses the need for alternative treatment approaches, for example, by focusing on cross-disorder traits, e.g., compulsive behavior, so treatment can be tailored to specific symptom domains.

The development of treatment approaches that focus on compulsive behavior requires knowledge of the underlying neural circuits. Compulsive behavior has been associated with structural and functional abnormalities within the frontal cortico-striatal-thalamo-cortical circuits in humans, as demonstrated with magnetic resonance imaging (MRI) (Montigny et al. 2013; Figee et al. 2016). Recent findings have implicated that compulsive behavior may involve four frontal cortico-striatal-thalamo-cortical circuits, each consisting of different cortical and striatal components (van den Heuvel et al. 2016). Within these circuits, compulsive behavior may either be caused by hyperactivity within the striatal component or by a failure of top-down control of the frontal cortical regions over the striatal component (Fineberg et al. 2010). It has been theorized that this top-down cortical control is mediated by the neurotransmitter glutamate (Sesack et al. 2003). This points towards a relationship between compulsive behavior and altered glutamate concentrations, which is further supported by the high density of glutamate receptors in the frontostriatal circuits (Monaghan et al. 1985) and dysregulation of glutamatergic signaling in individuals with ASD and OCD (Pittenger et al. 2011; Naaijen et al. 2015). In addition, the dopaminergic system has a regulatory role in the activity within the cortico-striatal-thalamo-cortical circuits (Haber 2016). Dopaminergic stimulation through chemogenetics has been shown to increase glutamate levels in the frontal cortex and striatum (Casado-Sainz et al. 2022). These results signify the tight interaction between dopaminergic and glutamatergic transmission in the cortico-striatal-thalamo-cortical circuits, and its potential involvement in compulsivity.

The role of glutamate in patients with OCD and ASD may imply that anti-glutamatergic drugs could be effective as medication against compulsivity (Mechler et al. 2017). For example, N-acetylcysteine has shown initial promise to reduce compulsive behavior in humans (Oliver et al. 2015). Another potential drug is the *N*-methyl-d-aspartate (NMDA) receptor antagonist memantine, an FDA-approved drug used in the clinic for the symptomatic treatment of Alzheimer’s disease (see for a systematic review and meta-analysis: Matsunaga et al., 2015). Memantine is an open-channel NMDA receptor blocker with rapid response kinetics (Chen et al. 1992), which may protect neurons against glutamate excitotoxicity with limited side effects (Rammes et al. 2008). Memantine has shown some initial promise as a successful treatment against OCD symptoms in human adults with OCD. As an add-on treatment in human adults with OCD, memantine was found to reduce the severity of symptoms (Ghaleiha et al. 2013; Haghighi et al. 2013). However, three-quarters of people with OCD experience their first symptoms in mid-childhood (Boileau 2011). Therefore, it is important to assess the treatment potential of memantine in children and adolescents (Mechler et al. 2017). In addition, mechanistically, it remains unclear how memantine exerts its therapeutic effects on neural circuits involved in OCD.

Therefore, the objective of this study was to determine the therapeutic efficacy of memantine in the reduction of compulsive behavior during adolescence. Because of the presence of dopaminergic abnormalities in individuals with OCD (Denys et al. 2004) and the tight interaction between the glutamatergic and dopaminergic systems in the brain, we used a dopamine-based animal model of compulsivity. To that aim, we measured the behavioral effects of memantine administration in adolescent rats with quinpirole-induced compulsive checking behavior (Straathof et al. 2020). In addition, we aimed to elucidate memantine’s possible mode of action on the development of structural and functional connectivity and functional activation within the frontostriatal system, which we measured with structural and functional MRI methods.

## Methods

All experiments were approved by the Committee for Animal Experiments of the University Medical Center Utrecht, The Netherlands (2014.I.12.104). All efforts were made to reduce the number of animals used and minimize animal suffering.

### Animal model

We used a recently described adolescent rat model of compulsive checking behavior (Straathof et al. 2020), adapted from an established adult rat model of compulsive checking behavior (Szechtman et al. 1998).

Sixty-four juvenile male Sprague–Dawley rats (Harlan, the Netherlands) were housed individually and habituated to environmental conditions (temperature 22–24 °C and 12-h light/dark cycle with lights on at 7:00 AM) for at least 7 days prior to the experiment, with access to food and water ad libitum. From the age of 5 weeks (body weight: 105 ± 18 g (mean ± standard deviation (SD)), corresponding to puberty (Sengupta 2013), we subcutaneously injected the rats with the selective D2/D3 receptor agonist quinpirole (Tocris, UK, 0.5 mg/kg; *n* = 32; compulsive group) or saline (*n* = 32; control group), twice per week during 6 weeks (total of 12 injections). We randomly assigned the treatment (quinpirole or saline) to the animals. The experimenters (CvH, CES, MS, and ELAB) could not be blinded for the factor group (compulsive or control), due to the obvious behavioral effects of quinpirole treatment. Nevertheless, the experimenters were blinded for memantine or saline treatment. Each injection was immediately followed by placing the rat in the center of a large open field table (160 × 160 cm, 60 cm above the floor) for 30 min. On the open field table, four objects (2 black, 2 white; 8 × 8 × 8 cm) were placed in fixed locations: two near the middle and two near the corners of the table. The combination of repetitive quinpirole injections and placement on the open field is essential for developing compulsive checking behavior (Szechtman et al. 1998).

### Experimental groups

The potency of memantine treatment to reduce compulsive behavior and its mode of action in the quinpirole-induced adolescent rat model of compulsive checking behavior was assessed in two studies.

In study I, we measured the effects of a sub-chronic memantine treatment on compulsive behavior and structural and functional connectivity in the frontostriatal system of adolescent rats. Rats were randomly assigned to memantine or saline treatment, and the experimenters were blinded for this treatment assignment. Rats received daily intraperitoneal injections of the NMDA receptor antagonist memantine (20 mg/kg/day (Sekar et al. 2013), Boehringer Ingelheim Pharma, Germany) (compulsive + memantine group: *n* = 8, control + memantine group: *n* = 8) or saline (compulsive + saline group: *n* = 8, control + saline group: *n* = 8) for seven consecutive days, starting the day after the 10th quinpirole or saline injection. On days when both injections were given (11th and 12th quinpirole/saline injection), the memantine/saline treatment was given 30 min before the quinpirole/saline injection.

In study II, we measured the acute effects of a single memantine injection on functional activation in the frontostriatal system. Rats received an acute intravenous memantine (20 mg/kg, Sigma-Aldrich, Germany; compulsive + memantine: *n* = 12, control + memantine: *n* = 12) or saline injection (compulsive + saline: *n* = 4, control + saline group: *n* = 4) 130 min after the 12th quinpirole/saline injection during pharmacological MRI. Groups receiving saline during MRI were smaller because our previous study showed no significant effects of saline injections on pharmacological MRI (Roelofs et al. 2017). We included an extra experimental group of rats to assess the influence of possible pharmacological interactions between memantine and quinpirole. In this group, rats (*n* = 8) received only one quinpirole injection, followed by a single memantine injection 130 min after quinpirole injection during pharmacological MRI.

### Behavioral analyses

Ethovision software (Noldus Information Technology B.V., the Netherlands) was used to automatically trace the locomotor trajectories of the rats on the open field table on the days of the MRI experiments. The open field area was virtually divided into 25 rectangles of 40 × 40 cm^2^ of which the outer zones extended 20 cm outside the open field. For all analyses, we used the last 15 min for the compulsive rats, and the complete 30 min for the control rats (Straathof et al. 2020). Since the effect of quinpirole is biphasic, i.e., a short inhibition period is followed by extensive excitation, we only included the second half of the observation period for behavioral assessment of the quinpirole rats. However, because control rats usually show little activity in this second half of the observation period, the whole observation period was used for the assessment of control rats. We calculated the frequency of visits for each zone during the observation period and defined the home base as the most frequently visited zone.

We quantified compulsive-like behavior of checking places/objects before and after memantine treatment in study I (after the 10th and 12th quinpirole/saline injections), and before the single memantine injection in study II (after the 12th quinpirole/saline injection). Compulsive checking behavior parameters were characterized relative to the home base and included frequency of checking (number of visits to the home base per minute, observed during the observation period), length of checks (average time of a visit at the home base), recurrence time of checking (average time spent in other areas before returning to the home base), and stops before returning to the home base (average number of other areas the rat visited before returning to the home base) (Szechtman et al. 1998; Tucci et al. 2014). In addition, we determined the predictability of the visited zones as the Lempel–Ziv source entropy (Song et al. 2010), using a maximal substring of three zones and only including animals that visited at least nine different zones.

To study the effects of memantine treatment on behavioral measures other than compulsive-like checking behavior, we performed additional behavioral measurements on the rats in study I. We calculated hyperactivity measures, including the total traveled distance, average movement velocity, and immobility time (< 0.01 cm movement per video frame). In addition, we manually quantified stereotypic behaviors the rats showed during a stop at their home base (Szechtman et al. 1998; Straathof et al. 2020), for the first twenty visits during the observation period. Because control rats were less active, all visits were included when the total amount of home-base visits was below twenty. First, we scored the entering or leaving direction relative to the home base for each visit to determine a potential directional preference. We used a compass divided into eight different directions (per 45 degrees) to determine the directions. Second, to quantify the horizontal movements per visit, we counted the number of anti-clockwise and clockwise turns. For vertical movements, we counted the number of head dips per visit. Third, the placement of the forelimbs and number of sniffs at the object were counted to score the interaction of the rat with the object per visit. Fourth, we determined the grooming time per visit. Lastly, these individual behavioral scores were combined into a total number of behavioral acts per visit.

### MRI acquisition

All MRI experiments were conducted on a 9.4 T MR system equipped with a 400 mT/m gradient coil (Varian, Palo Alto, CA, USA). A homebuilt 90-mm diameter Helmholtz volume coil was used for signal excitation and an inductively coupled 25-mm diameter surface coil for signal detection. On the days MRI was executed, rats were directly transferred from the open field test platform to the MRI scanner. Rats were anesthetized and endotracheally intubated for mechanical ventilation with 2% isoflurane in a mixture of air and O_2_ (70%/30%). Rats were subsequently immobilized in a specially designed stereotactic holder and placed in an animal cradle. One tail vein was cannulated under isoflurane anesthesia before placing the rat in the stereotactic holder for memantine or saline administration during MRI in study II. During MRI, end-tidal CO_2_ was continuously monitored with a capnograph (Microcap, Oridion Medical 1987 Ltd., Jerusalem, Israel) and body temperature was maintained at 37.0 ± 1.0 °C. Heart rate and blood oxygen saturation were monitored with an infrared sensor attached to the hind paw. Parameter settings for the MRI acquisitions were as followed:

- Anatomical MRI: 3D balanced steady-state free precession (BSSFP) scan with four-phase cycling angles (0°, 90°, 180°, 270°). Repetition time (TR)/echo time (TE) = 5/2.5 ms; flip angle = 20°; field-of-view (FOV) = 40 × 32 × 24 mm^3^; acquisition matrix = 160 × 128 × 96; image resolution = 250-μm isotropic. Total acquisition time = 12.5 min. The isoflurane anesthesia level was reduced to 1.5% at the start of the anatomical MRI acquisition to lower the anesthetic depth for the following resting-state fMRI or pharmacological MRI acquisition.
- Resting-state functional MRI: T_2_^*^-weighted blood oxygenation level-dependent (BOLD) images were acquired using a single-shot 3D gradient echo-planar imaging (EPI) sequence. TR/TE = 26.1/15 ms; FOV = 32.4 × 32.4 × 16.8 mm^3^; flip angle = 13°; acquisition matrix = 54 × 54 × 28; image resolution = 600-μm isotropic. Acquisition time = 730.8 ms per scan volume, with a total of 800 volumes resulting in a total scan time of 9 min and 45 s. Resting-state fMRI was always started at 90 min after quinpirole injection, to standardize the effects of quinpirole across animals.
- Diffusion-weighted MRI: 2D 4-shot spin echo EPI sequence: TR/TE = 1700/34 ms; FOV = 32 × 32 mm^2^; acquisition matrix = 64 × 128; 25 slices of 0.5 mm, image resolution = 500 × 250 × 500 μm^3^, zero-filled to 250 × 250 × 500 μm^3^; *b* = 1611 s/mm^2^; δ/Δ = 6.5/10.27 ms. Five non-diffusion-weighted (b0) and sixty diffusion-weighted images were acquired. Diffusion-weighted MRI was performed at 2% isoflurane anesthesia to minimize animal motion.
- Pharmacological MRI: 2D gradient-echo multi-slice sequence: TR/TE = 500/15 ms; FOV = 32 × 32 mm^2^; flip angle = 50°; acquisition matrix = 128 × 128; 25 slices of 0.5 mm, image resolution = 250 × 250 × 500 μm^3^; acquisition time = 128 s per scan volume, with a total of 47 volumes resulting in a total scan time of 103 min. Nine baseline scans were acquired, followed by an intravenous memantine or saline injection during the tenth scan and 37 post-injection scans.

For study I, MRI acquisitions were made after the 10th (pre-treatment measurement) and 12th (post-treatment measurement) injection of quinpirole/saline. The MRI session consisted of anatomical MRI, followed by resting-state fMRI acquisition and diffusion-weighted MRI. For study II, MRI acquisition was made after the 12th injection of quinpirole/saline. The MRI session consisted of anatomical MRI, followed by pharmacological MRI.

### MRI processing

Unless otherwise stated, analyses were performed using FMRIB’s Software Library (FSL) v5.0.9.

#### Regions of interest

Regions-of-interest were taken from the 3D rendering of the Paxinos and Watson atlas (Paxinos and Watson 2005). Regions included the frontal cortex (consisting of the orbitofrontal cortex (OFC: dorsolateral, lateral, medial, and ventral orbital cortex), the anterior cingulate cortex (ACC: cingulate cortex areas 1 and 2), and the medial prefrontal cortex (mPFC: prelimbic and infralimbic cortex)) and the striatum (caudate putamen (CPu) and nucleus accumbens (NAcc)) for all MR analyses. We measured interhemispheric homologous connectivity for these regions as well as frontostriatal intrahemispheric connectivity. Homotopic areas in the left and right hemispheres were combined for the pharmacological MRI analyses. In addition, resting-state fMRI and pharmacological MRI analyses were also performed for the different sub-regions (OFC, ACC, MPFC, CPu, & NAcc) separately.

#### Registration

We linearly registered individual anatomical images to a three-dimensional model of the Paxinos and Watson atlas (Paxinos and Watson 2005) and created a study-specific template by taking the mean of these registered images. Individual mean resting-state fMRI scans were linearly registered to the individual anatomical scan using *FLIRT* (Jenkinson and Smith 2001; Jenkinson et al. 2002), followed by non-linear registration to the study-specific anatomical template using *FNIRT* (Andersson et al. 2007). Individual averaged non-diffusion-weighted (b0) images were non-linearly registered to the average b0 image of one individual rat (DWI template), followed by linear registration to the study-specific anatomical template. Individual pharmacological MR images were directly non-linearly registered to the study-specific anatomical template. Regions-of-interest were transformed into individual space with the inverse of these registrations. The resting-state fMRI regions-of-interest were masked with a temporal signal-to-noise ratio mask of 10 and the regions-of-interest for DWI-based tractography were masked with a gray matter mask (fractional anisotropy (FA) lower than 0.25).

#### Diffusion-weighted MRI—study I

The diffusion-weighted images were brain extracted with *BET* (Smith 2002), motion, and eddy current corrected with affine transformations in *MCFLIRT* (Jenkinson et al. 2002), and the diffusion tensor was fitted using *dtifit* within the FMRIB’s Diffusion Toolbox (FDT package). Whole-brain tractography was performed using MrTrix3® (www.mrtrix.org (Tournier et al. 2012). The response function estimation for single-shell constrained spherical deconvolution (CSD) tractography was performed on individual datasets with a reimplementation of the tax method for response function estimation (Tax et al. 2014). Subsequently, the individual response functions were averaged to obtain a group response function. We performed whole-brain CSD tractography on individual datasets with one million streamlines, and streamlines-of-interest were selected using our regions-of-interest as start- and endpoints. The median fractional anisotropy (FA), reflecting the degree of diffusion anisotropy (degree of restricted diffusion along with the main directions of the diffusion tensor), was used as a measure of structural connectivity (Koay et al. 2006).

#### Resting-state fMRI—study I

Preprocessing steps of the resting-state fMRI scans included removing the first 20 images to reach a steady state, motion correction, brain extraction, and removal of noise components with single-subject independent component analysis (Beckmann and Smith 2004). Moreover, the BOLD signal was normalized, and motion correction parameters were used as regressors for the resting-state signal. Low-frequency BOLD fluctuations were obtained by applying temporal filtering between 0.01 and 0.1 Hz in AFNI (Cox 1996). We calculated Fisher’s *Z*-transformed correlation coefficients for inter- and intrahemispheric functional connectivity between regions-of-interest.

#### Pharmacological MRI—study II

Preprocessing steps of the pharmacological MRI scans included removing the first baseline scan, brain extraction, and motion correction. The BOLD response to the memantine/saline injection was normalized to the mean baseline signal (mean of the first 8 images). To calculate brain activation maps, we used a repeating OFF/ON design as a regressor for a voxel-wise generalized linear model (GLM) per group, in which “OFF” corresponded with the pre-injection scans and “ON” with the first 25 post-injection scans. The resulting *z* activation maps per group were false discovery rate (FDR) corrected, with a threshold at *z* = 3.1 corresponding to *p* < 0.001 after FDR correction The brain activation response to memantine or saline injection was calculated as the positive area under the curve (AUC) of the BOLD signal time course for each individual rat (negative values were excluded). This AUC represents the percentage of BOLD signal change per second.

### Statistics

Statistical analyses were performed in R (3.2.3) and Rstudio 0.99.903 (R Core Team 2014). *P*-values were FDR corrected and considered significant below 0.05 after FDR correction.

Differences in compulsive behavioral metrics before memantine treatment (study I and II separately) between the compulsive and control group were analyzed with a Mann–Whitney *U* test, to verify that compulsive-like behavior developed before memantine injections.

For study I, we assessed the interaction between treatment (saline vs. memantine) and group (control vs. compulsive) for various types of behavior (compulsive checking behavior, hyperactivity measures, and behavior during stops), body weight, functional connectivity, and structural connectivity with a mixed-design ANOVA, with factor “time” as a within-subject variable, and factors “group” (control or compulsive) and “treatment” (saline or memantine) as between-subject variables. The main effect of interest in these analyses is the time*group*treatment interaction, showing whether saline and memantine have different effects on the control and compulsive groups. *P*-values were FDR corrected per modality in the behavior, functional, and structural connectivity analyses. In addition, we compared pre- and post-measurements for each group separately with Wilcoxon signed-rank tests to determine the saline treatment effects specifically.

For statistical analysis of pharmacological MRI data in study II, we compared the BOLD activation responses (AUC) between groups using a Kruskal–Wallis test, followed by post hoc Dunn’s tests, adjusted for multiple comparisons using the Benjamini Yekutieli method for FDR correction (Benjamini and Yekutieli 2001).

## Results

In study I, one control rat died during the post-memantine MRI acquisition because of respiratory problems caused by excessive mucus. In addition, the behavioral recording of one compulsive rat was incomplete and the MRI scans of one control and one compulsive rat were affected by artifacts. Therefore, final groups in study I consisted of fourteen compulsive rats (compulsive + saline treatment: *n* = 7; compulsive + memantine treatment: *n* = 7) and fourteen control rats (control + saline treatment: *n* = 8; control + memantine treatment: *n* = 6).

In study II, all included animals could be used for analyses, resulting in final group sizes of sixteen compulsive rats (compulsive + saline injection: *n* = 4; compulsive + memantine injection: *n* = 12) and sixteen control rats (control + saline injection: *n* = 4; control + memantine injection: *n* = 12).

### Study I: Memantine treatment does not reduce compulsive behavior and frontostriatal structural and functional connectivity in adolescent rats

Before memantine treatment in study I or memantine injection in study II, compulsive rats displayed clear patterns of repeated traveling between two zones of the open field and compulsive-like checking behavior (Supplementary Fig S1).

**Fig. 1:**
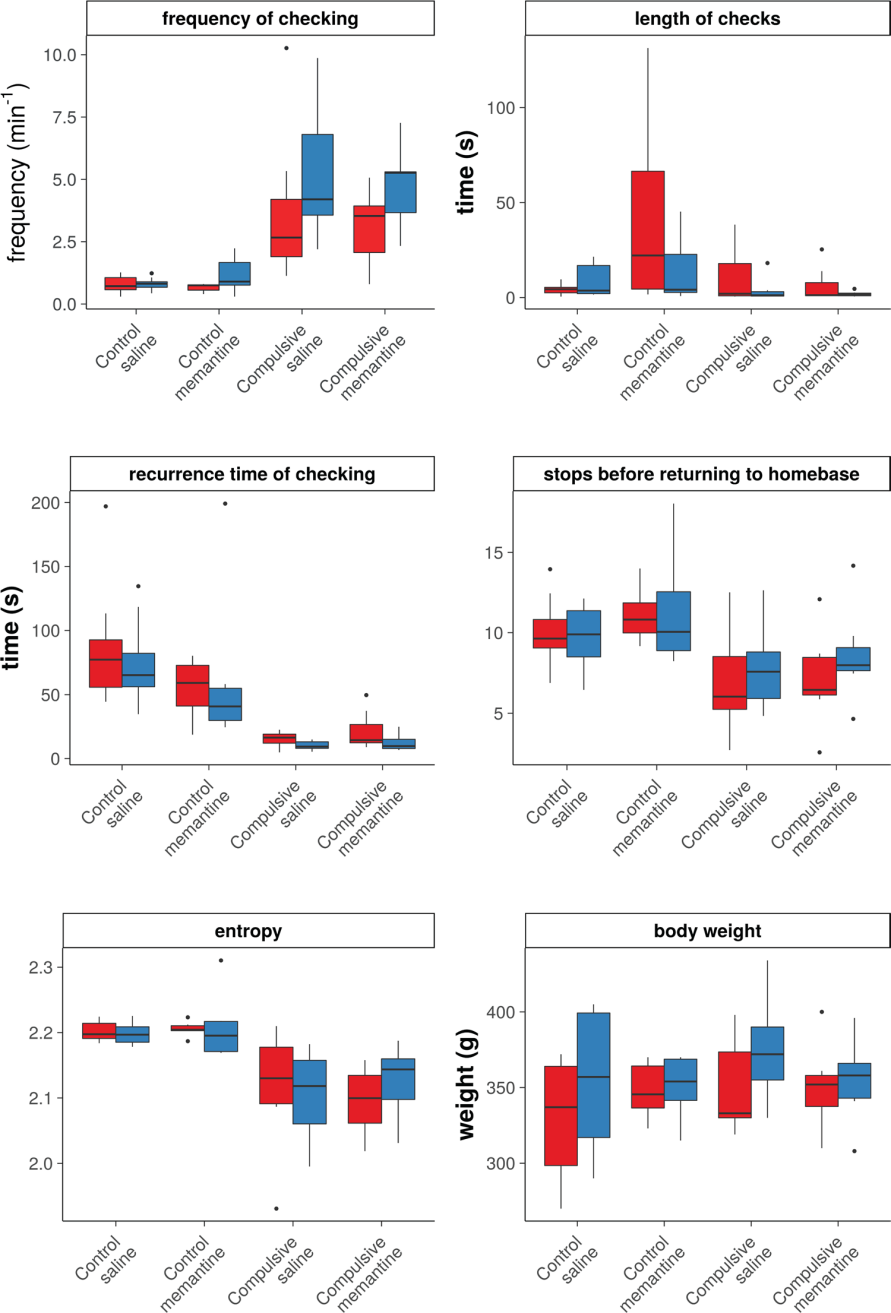
Measures of compulsive checking behavior and body weight, before, and after saline/memantine treatment in control and compulsive rats. Compulsive behavior measures (frequency of checking (number of visits at the home base per minute (observed during 15 min for compulsive rats, and during 30 min for controls)), length of checks (average time (s) spent at the home base), recurrence time of checking (average time (s) before returning to the home base), stops before returning to the home base (average number of zones visited in between two visits of the home base)), entropy (predictability of the visited zones), and body weight (g), before (red), and after (blue) 7 days of daily saline/memantine treatment (control + saline: *n* = 8; control + memantine: *n* = 6; compulsive + saline: *n* = 7; compulsive + memantine: *n* = 7). Error bars represent 1.5 times the interquartile range, and dots represent values that exceeded 1.5 times the interquartile range

**Fig. 2:**
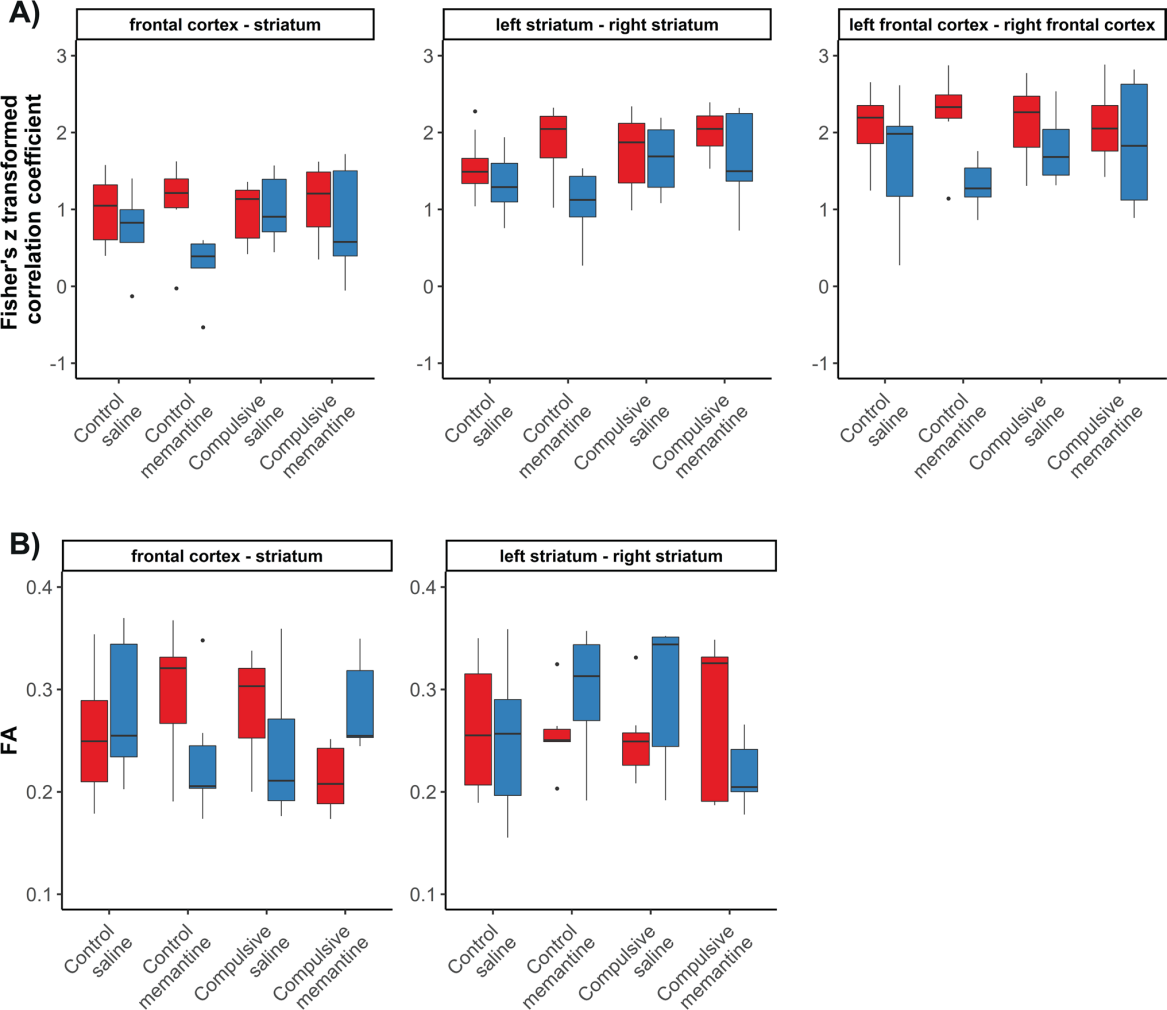
Functional and structural connectivity in the frontostriatal system before and after saline/memantine treatment in control and compulsive rats. Bar graphs of functional connectivity (Fisher’s Z-transformed correlation coefficient) (**A**) and structural connectivity (median fractional anisotropy (FA)) (**B**) of intra- and interhemispheric connections within the frontostriatal system before (red) and after (blue) 7 days of daily saline/memantine treatment (control + saline: *n* = 8; control + memantine: *n* = 6; compulsive + saline: *n* = 7; compulsive + memantine: *n* = 7). Structural connectivity between the left and right frontal cortex could not be determined because of unreliable tractography results. Error bars represent 1.5 times the interquartile range and dots represent values that exceed 1.5 times the interquartile range

We did not find significant interaction effects between factors “time” (pre- or post-treatment), “group” (compulsive or control) and “treatment” (saline or memantine) for any of the compulsive behavioral measures (Fig. 1; time*group*treatment effects: frequency of checking: *F*(1,24) = 0.44 (*F*(degrees of freedom numerator, degrees of freedom denominator)); *p* = 0.66; length of checks: *F*(1,24) = 4.40; *p* = 0.40; recurrence time of checking: *F*(1,24) = 1.14; *p* = 0.54; stops before returning to the home base: *F*(1,24) = 0001; *p* = 0.97; entropy: *F*(1,24) = 0.51; *p* = 0.66). For statistical results of factor and interaction effects on all behavioral measures, please see Supplementary Table 1. In our additional analyses comparing pre- and post-treatment measurements specifically for each group, we detected no statistically significant effect of saline treatment on any of the compulsive behavioral measures, in both the compulsive and control groups (Fig. 1; Supplementary Table 2). Comparable results were found for the additional behavioral measures, including hyperactivity measures and stereotypic behaviors during stops at the home base (Supplementary Fig. S2; Supplementary Tables 1 and 2). Correspondingly, we also did not find statistically significant interactions effects between group, time and treatment on functional or structural connectivity in the frontostriatal system (Fig. 2; functional connectivity: time*group*treatment effects: frontal cortex–striatum: *F*(1,24) = 0.26; *p* = 0.61; left–right frontal cortex: *F*(1,24) = 1.14; *p* = 0.61; left–right striatum: *F*(1,24) = 0.70; *p* = 0.61; structural connectivity: time*group*treatment effects: frontal cortex–striatum: *F*(1,24) = 0.62; *p* = 0.44; left–right striatum: *F*(1,24) = 1.39; *p* = 0.44) (see Supplementary Tables 3 and 4 for statistical results on functional connectivity and structural connectivity, respectively). Similarly, functional connectivity analyses on individual sub-regions of the frontal cortex and striatum did not reveal statistically significant effects of memantine treatment (Supplementary Fig. S3).

We found a significant interaction effect between factors “time” and “treatment” (saline or memantine) on body weight (*F*(1,24) = 29.30; *p* = 0.0003). Body weight of control and compulsive adolescent rats increased in 7 days between pre- and post-treatment with saline (control: pre: 330 ± 38 g; post: 355 ± 45 g, *p* = 0.01 before FDR correction; quinpirole: pre: 351 ± 31 g; post: 375 ± 34 g, *p* = 0.01 before FDR correction) (Fig. 1), but not in rats that were treated with memantine.

### Study II: Memantine activates the frontal cortex, but not after quinpirole injection in adolescent rats

**Fig. 3:**
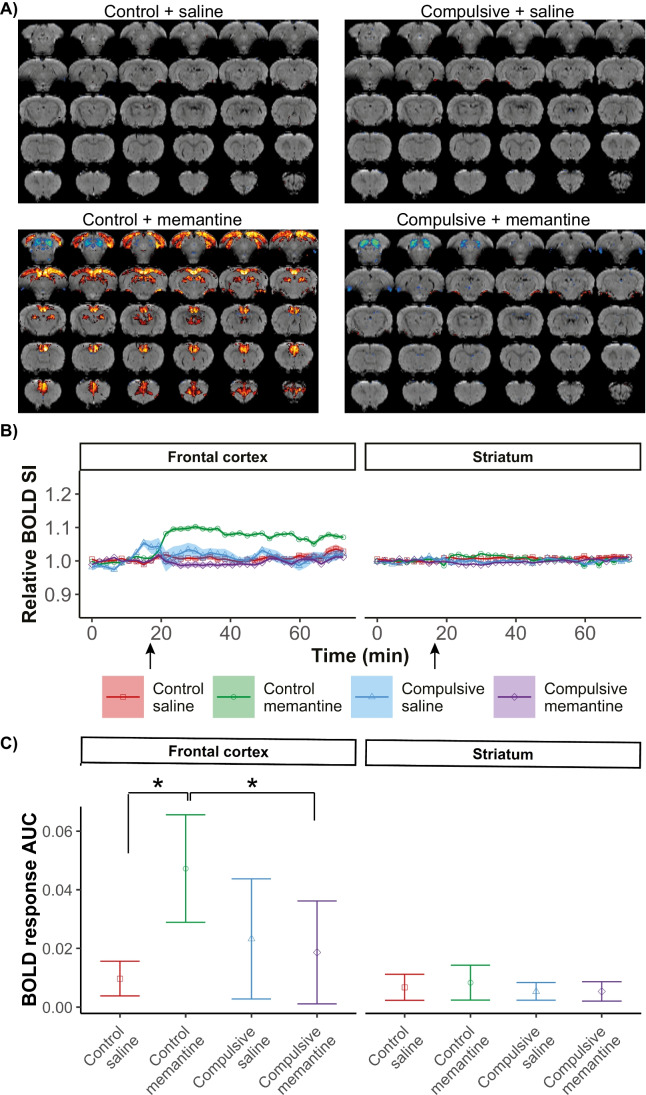
Brain activation directly after memantine/saline injection in control and compulsive rats. Brain activation maps, overlaid on anatomical images, show positive BOLD activation responses in yellow/red (z > 3.1) and negative responses in blue (z <  − 3.1) (**A**). The normalized BOLD signal intensity (SI) time course is shown as averaged time series for the regions-of-interest, with the arrow indicating the time of memantine/saline injection (**B**). BOLD responses to memantine or saline injection quantified as area under the curve (AUC) (relative positive BOLD SI change per second) (**C**). Control + saline: *n* = 4; control + memantine: *n* = 12; compulsive + saline: *n* = 4; compulsive + memantine: *n* = 12. *Corrected *p* < 0.05. Shades in B represent the standard error. Error bars in C represent the standard deviation

Figure 3A shows brain activation maps, which display regions where memantine/saline injection resulted in a significant activation response. As expected, the saline injection did not activate brain areas in control nor compulsive rats, as also illustrated from BOLD signal time courses in the frontal cortex and striatum (Fig. 3B). However, memantine injection-induced clear positive brain activation in frontal and occipital cortical areas in control rats, but not in compulsive rats. We detected a significant group effect (H(3) = 7.601; *p* = 0.0007) in the frontal cortex, but not in the striatum (H(3) = 0.96; *p* = 0.425).

Post hoc analyses demonstrated that in control rats, the AUC of the BOLD response in the frontal cortex was significantly higher after memantine injection than saline injection (memantine: AUC = 0.05 ± 0.02; saline: AUC = 0.01 ± 0.01, *p* = 0.004) (Fig. 3C). In compulsive rats, the AUCs were similar between memantine- and saline-injected rats for all measured areas. Memantine-induced BOLD responses in the frontal cortex were statistically significantly higher in control than in compulsive rats (control: AUC = 0.05 ± 0.02; compulsive: AUC = 0.02 ± 0.02, *p* = 0.002). The AUCs for the striatum after memantine or saline injection were similar between groups. We found similar results for analyses of the separate sub-regions of the frontal cortex (ACC, OFC, mPFC) and striatum (CPu and NAcc) (Supplementary Fig. S4).

**Fig. 4:**
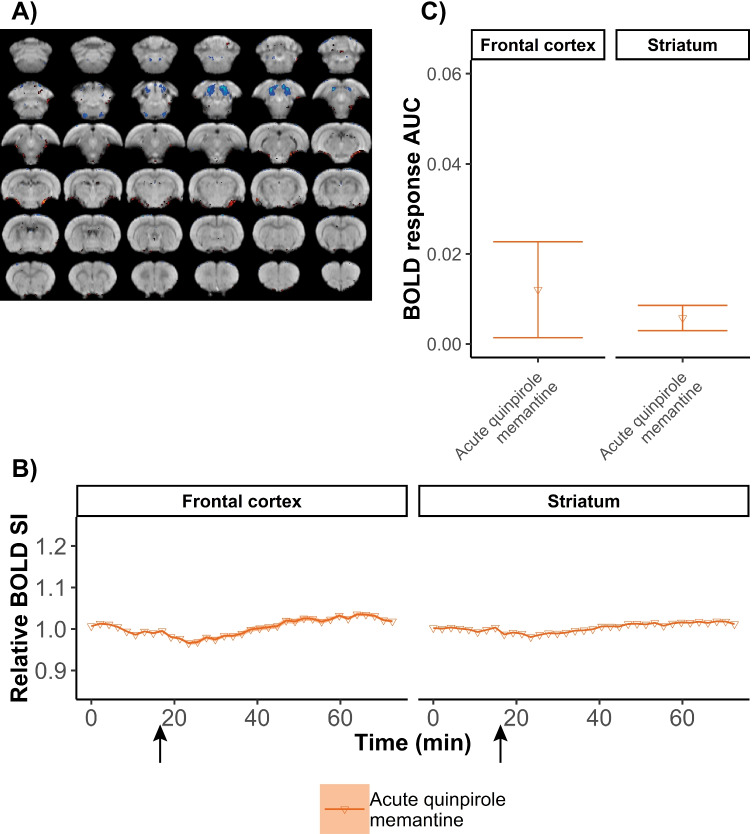
Brain activation directly after memantine injection following a single-quinpirole injection. Brain activation maps, overlaid on anatomical images, show positive BOLD activation responses in yellow/red (z > 3.1) and negative responses in blue (z <  − 3.1) (**A**). The normalized BOLD signal intensity (SI) time-course is shown as averaged time series for the regions-of-interest, with the arrow indicating the time of memantine injection (**B**). BOLD responses to memantine injection quantified as area under the curve (AUC) (relative positive BOLD SI change per second) (**C**). Acute quinpirole + memantine: *n* = 8. Shades in B represent the standard error. Error bars in C represent the standard deviation

Lastly, to assess the influence of possible pharmacological interaction between memantine and quinpirole, we included an additional experimental group. Each rat received only a single-quinpirole injection, followed by a single memantine injection. Quantitative assessment revealed no statistically significant differences in the AUC of the BOLD response in the frontal cortex and striatum between compulsive and single-quinpirole-injected rats (Fig. 4).

## Discussion

In this study, we determined the therapeutic efficacy of memantine to reduce developed compulsive-like behavior in adolescent rats and assessed its possible mode of action on the frontostriatal system (study I). We showed that a repeated memantine treatment regimen did not reduce compulsive-like behavior in the adolescent rat model of quinpirole-induced compulsive checking behavior. Correspondingly, memantine treatment did not induce structural and functional connectivity changes in the frontostriatal system. A single-memantine injection activated frontal cortical regions in control but not in compulsive adolescent rats or rats that received a single-quinpirole injection (study II), which may explain the absence of memantine-induced treatment effects in study I.

The main aim of our study was to identify and elucidate possible therapeutic effects of the NMDA receptor antagonist memantine in adolescent compulsive behavior (study I). Several glutamatergic antagonists effectively reduce OCD symptoms in adults (Pittenger et al. 2011) and ASD-associated behavior in pediatric patients (Mechler et al. 2017). Before memantine treatment, quinpirole-injected rats demonstrated clear compulsive-like checking behavior, similar to our earlier study in the adolescent model (Straathof et al. 2020) and comparable to the adult model (Szechtman et al. 1998). In line with clinical treatment regimes, we started the memantine treatment after developing compulsive checking behavior (i.e., after the 10th quinpirole/saline injection). The repetitive quinpirole injections were continued during the memantine treatment period, to ensure the persistence of quinpirole-induced compulsive behavior (de Haas et al. 2011). However, the applied memantine treatment regimen did not reduce compulsive-like behavior and functional or structural connectivity in the frontostriatal circuitry. Similar to findings in OCD patients, varying results have been reported about the ability of pharmacological agents to reduce compulsive behavior in 3s, including quinpirole-injected adult rats (Stuchlik et al. 2016). The absence of a memantine treatment effect may be explained by the dosing regimen, administration time, and the administered dose of memantine. The administered dose was based on a previous subchronic memantine treatment study in rats (Sekar et al. 2013). We are confident that this memantine dose is effective in our rats since it significantly influences body weight. Nevertheless, the applied dose and treatment regime may not effectively reduce quinpirole-induced compulsive behavior, and future dose–response studies could investigate this. However another explanation could be that the treatment effects of memantine and quinpirole may have interacted since memantine and quinpirole can induce similar effects in the brain—for example, memantine and quinpirole both induce long-term depression in the striatum (Mancini et al. 2016).

To assess the effect of memantine on brain network activity, we measured direct activation responses with pharmacological MRI (study II). For this acute study, memantine was administered intravenously to achieve direct bioavailability, enabling rapid scanning. Although this results in different drug delivery as compared to the intraperitoneal administration in study I (Lee et al. 2016), we assume the working mechanisms of memantine would not be different. A previous study demonstrated that memantine can elicit increased and decreased activity in the prelimbic cortex in drug-naive rats (Sekar et al. 2013). Micro-dialysis studies have suggested that NMDA-receptor antagonists can activate prefrontal areas by increasing the glutamatergic tone (Moghaddam et al. 1997; López-Gil et al. 2007), which can activate other glutamatergic receptors. We found that memantine injection in control rats not only influences brain activity in the frontal cortex, but also results in activation of remote brain areas. However, this memantine-induced activation was largely absent in quinpirole-induced compulsive rats or in rats that received only a single-quinpirole injection. This suggests that the absence of memantine-induced activation in the compulsive group in study II was not fundamentally associated with the compulsive-like phenotype by sensitization of dopamine D2/D3 receptors, but more likely a result of interactions between pathways activated by memantine and quinpirole. There is a tight interaction between glutamatergic and dopaminergic receptors in the brain (Cepeda et al. 2009). Around half of the neurons in the striatum receive dual input, from cortico-striatal glutamatergic projections and nigrostriatal dopaminergic projections (Garside et al. 1996). Dopaminergic projections towards D1 and D2 receptors in the striatum play a crucial role in modulating signaling in the basal ganglia, with D2 receptors mainly involved in the indirect pathway (Nair et al. 2014). Dopamine D2 agonists, like quinpirole, inhibit glutamate efflux in the striatum (Maura et al. 1988). In addition, quinpirole has been shown to attenuate the excitatory effects of NMDA and AMPA receptors in the prefrontal cortex (Tseng and O’Donnell 2004) and striatum (Cepeda et al. 1993). This attenuating effect of quinpirole on glutamatergic neurotransmission may explain the absence of memantine-induced prefrontal activation in quinpirole-injected adolescent rats. This suggests that anti-glutamatergic drugs may not be effective in individuals with altered dopaminergic neurotransmission. In addition, direct interactions between quinpirole and memantine may have occurred at the dopamine D2/D3 receptor level, because both substances are dopamine D2 receptor agonists (Seeman et al. 2008; Mancini et al. 2016). Although quinpirole and memantine may have directly interacted on days when both substances were administered (three times), memantine was also administered four times without the administration of quinpirole, leading to a sub-chronic treatment effect. Therefore, both direct and indirect interactions between quinpirole and memantine may have obscured the treatment effects of memantine on compulsive behavior in this adolescent rat model in study I.

A possible limitation of the current study is the use of isoflurane anesthesia during resting-state fMRI acquisition. It has been shown that anesthesia with a comparable level of isoflurane (1.3%) as used in our study (1.5%) leads to enhanced functional connectivity values in cortico-striatal connections and diminished functional connectivity values between subcortical regions, as compared to awake rats (Paasonen et al. 2018). Thus, isoflurane anesthesia-induced changes in cortico-striatal functional connectivity may have obscured the effects of quinpirole or memantine on functional connectivity in this circuitry. However, since all groups were scanned under the same anesthesia conditions, we expect that specific group differences in functional connectivity would still have been detectable. In addition, we applied the memantine treatment after the development of compulsive behavior, to mimic potential clinical application. Nevertheless, it would be interesting to study whether memantine may reduce the development of compulsive behavior, because of the tight interactions between the dopaminergic and glutamatergic systems in the brain. Lastly, we only tested the therapeutic efficacy of memantine treatment in male rats, because there is insufficient knowledge about the quinpirole model in females, and early-onset OCD is more common in males (Ruscio et al. 2010). Nevertheless, it is important to evaluate the therapeutic efficacy of drugs against OCD in both sexes.

In conclusion, our study did not reveal the beneficial effects of memantine treatment on quinpirole-induced compulsive checking behavior in adolescent rats. This absence of a memantine treatment effect may be partly explained by model-treatment interactions between the dopaminergic and glutamatergic systems. Future studies that apply this model should carefully consider possible interactions between quinpirole and pharmacological treatments, verified in parallel pharmacological MRI experiments.

## Supplementary Information

Below is the link to the electronic supplementary material.

Supplementary file1 (DOCX 1907 KB)

## Declarations

### Disclaimer

This paper reflects only the author’s views, and the European Union is not liable for any use that may be made of the information contained therein.
