# Supplementary material for: Memantine treatment does not affect compulsive behavior or frontostriatal connectivity in an adolescent rat model for quinpirole-induced compulsive checking behavior
Source: Psychopharmacology (Berl). 2022 Apr 14;239(8):2457–70. doi: 10.1007/s00213-022-06139-z (PMC9293859; doi:10.1007/s00213-022-06139-z)
Supplement: Supplementary file 1 — Supplementary file1 (DOCX 1907 KB) [file 213_2022_6139_MOESM1_ESM.docx]

**Memantine treatment does not affect compulsive behavior or frontostriatal connectivity in an adolescent rat model for quinpirole-induced compulsive checking behavior**

Milou Straathof ^1 *^, Erwin L.A. Blezer ^1^, Christel E. Smeele ^1^, Caroline van Heijningen ^1^, Annette van der Toorn ^1^, TACTICS Consortium ^2^, Jan K. Buitelaar ^3,4^, Jeffrey C. Glennon ^3,5^, Willem M. Otte ^1,6^ & Rick M. Dijkhuizen ^1 *^

*^1^ Biomedical MR Imaging and Spectroscopy Group, Center for Image Sciences, University Medical Center Utrecht & Utrecht University, the Netherlands.*

*^2^ Members of the TACTICS consortium are listed in the Acknowledgments.*

*^3^ Department of Cognitive Neuroscience, Donders Institute for Brain, Cognition and Behavior, Radboud University Medical Center, Nijmegen, the Netherlands.*

*^4^ Karakter Child and Adolescent Psychiatry University Center, Nijmegen, the Netherlands.*

*^5^ Conway Institute of Biomolecular and Biomedical Research, School of Medicine, University College Dublin, Dublin 4, Ireland.*

*^6^ Department of Pediatric Neurology, UMC Utrecht Brain Center, University Medical Center Utrecht and Utrecht University, the Netherlands.*

* Corresponding authors:

M. Straathof
Postal address: Heidelberglaan 100, 3584 CX Utrecht, the Netherlands

Phone: +31 (0) 30 253 5568

E-mail address: M.Straathof-2@umcutrecht.nl

R.M. Dijkhuizen

Postal address: Heidelberglaan 100, 3584 CX Utrecht, the Netherlands

Phone: +31 (0) 30 253 5569

E-mail address: R.M.Dijkhuizen@umcutrecht.nl

**
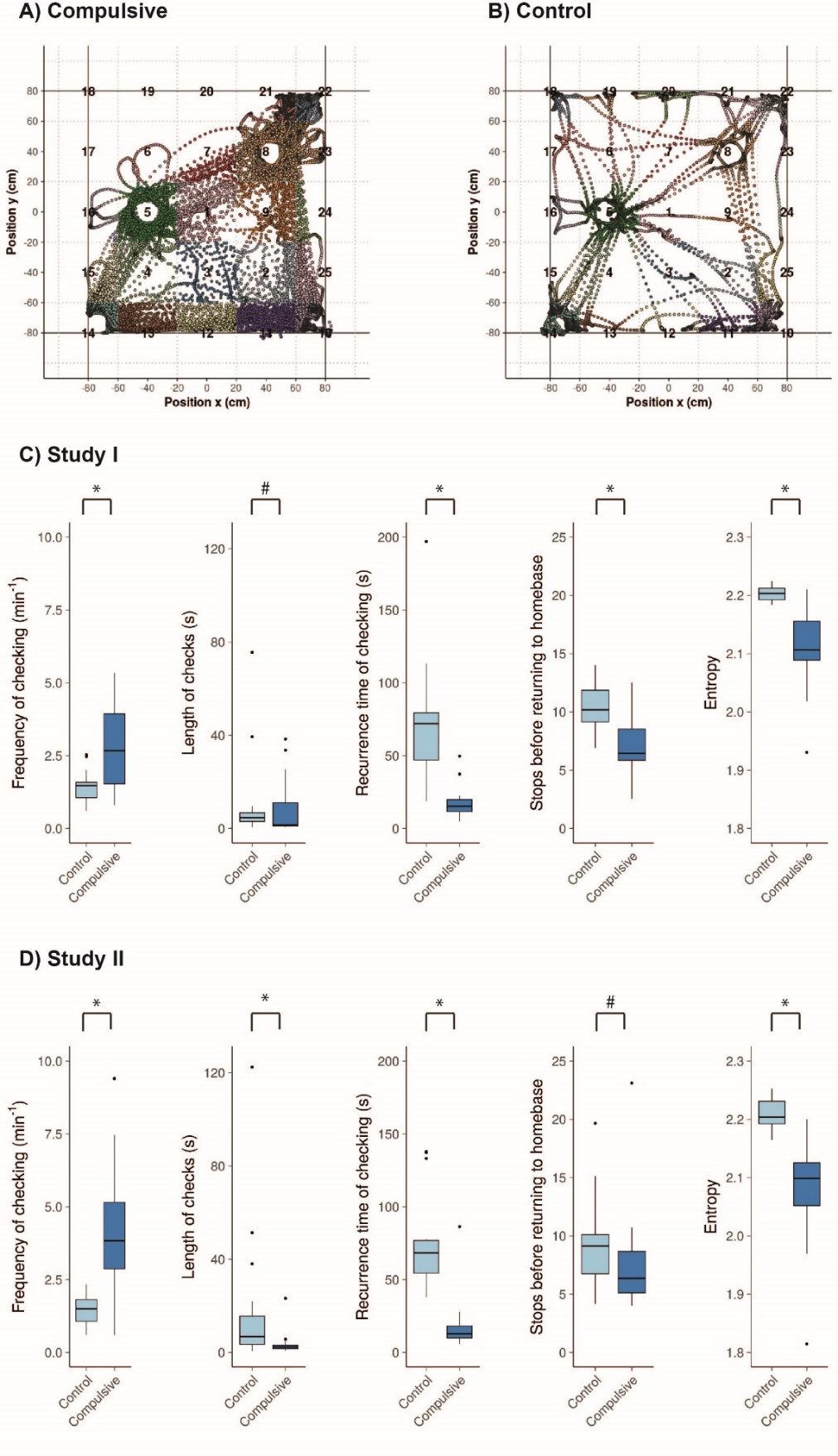
Supplementary Information**

**Supplementary Figure S1: Locomotor trajectories and behavioral metrics for control and compulsive adolescent rats before saline/memantine treatment.** A representative locomotor trajectory of a compulsive (A) and a control adolescent rat (B) during the observation period after the 10^th^ quinpirole/saline injection. The different zones on the open field are numbered, and locomotor trajectories are colored corresponding to these zones. We characterized behavior during the open field test for the last 15 minutes for compulsive rats and the full 30 minutes for control rats. Boxplots of the frequency of checking (number of visits at the home-base per minute (observed during 15 minutes for compulsive rats, and during 30 minutes for controls), length of checks (average time (s) spent at the home-base), recurrence time of checking (average time (s) before returning to the home-base), stops before returning to the home-base (average number of zones visited in between two visits of the home-base) and entropy (predictability of the visited zones) for control and compulsive rats prior to memantine/saline treatment in Study I (C, after the 10^th^ quinpirole/saline injection: Compulsive: n=14; Control: n=14) and prior to memantine/saline injection in Study II (D, after the 12^th^ quinpirole/saline injection: Compulsive: n=16; Control: n=16). * corrected p<0.05; # corrected p<0.1. Error bars represent 1.5 times the interquartile range, and dots represent values that exceeded 1.5 times the interquartile range.

**
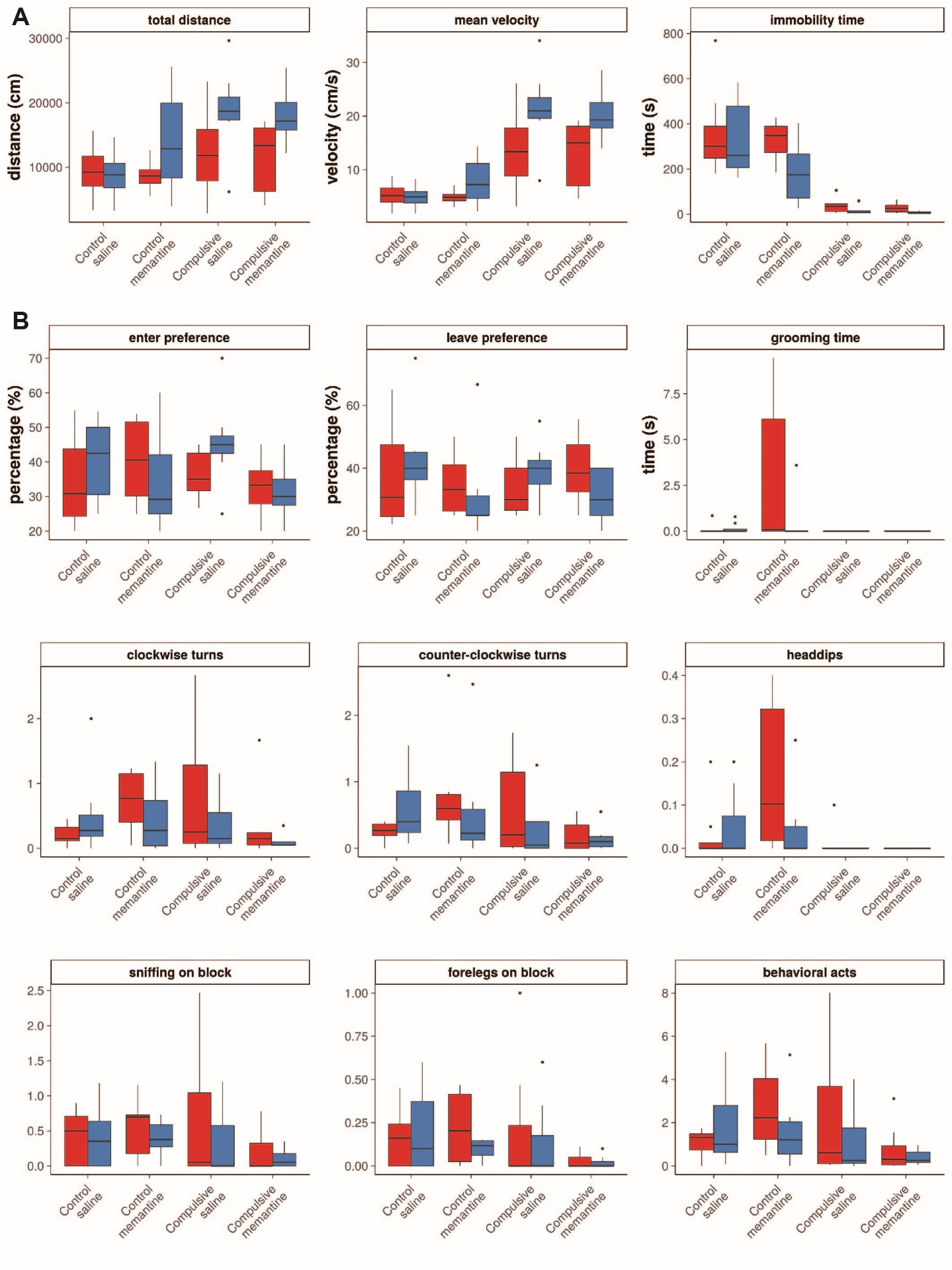
**

**Supplementary Figure S2: Measures of hyperactivity and stereotypic behavior before and after saline/memantine treatment in control and compulsive rats.** Hyperactivity measures (total distance moved, mean velocity and total immobility time (<0.01 cm movement per video frame)) (A) and stereotypic behaviors (enter preference (percentage of same enter direction of home-base zone), leave preference (percentage of same leave direction of home-base zone), average grooming time, number of clockwise turns, number of counter-clockwise turns, number of head dips, number of sniffs on block, number of placement of forelimbs on block, and total number of behavioral acts per home-base visit) (B) before (red) and after (blue) seven days of memantine/saline treatment. Error bars represent 1.5 times the interquartile range, and dots represent values that exceeded 1.5 times the interquartile range.

**
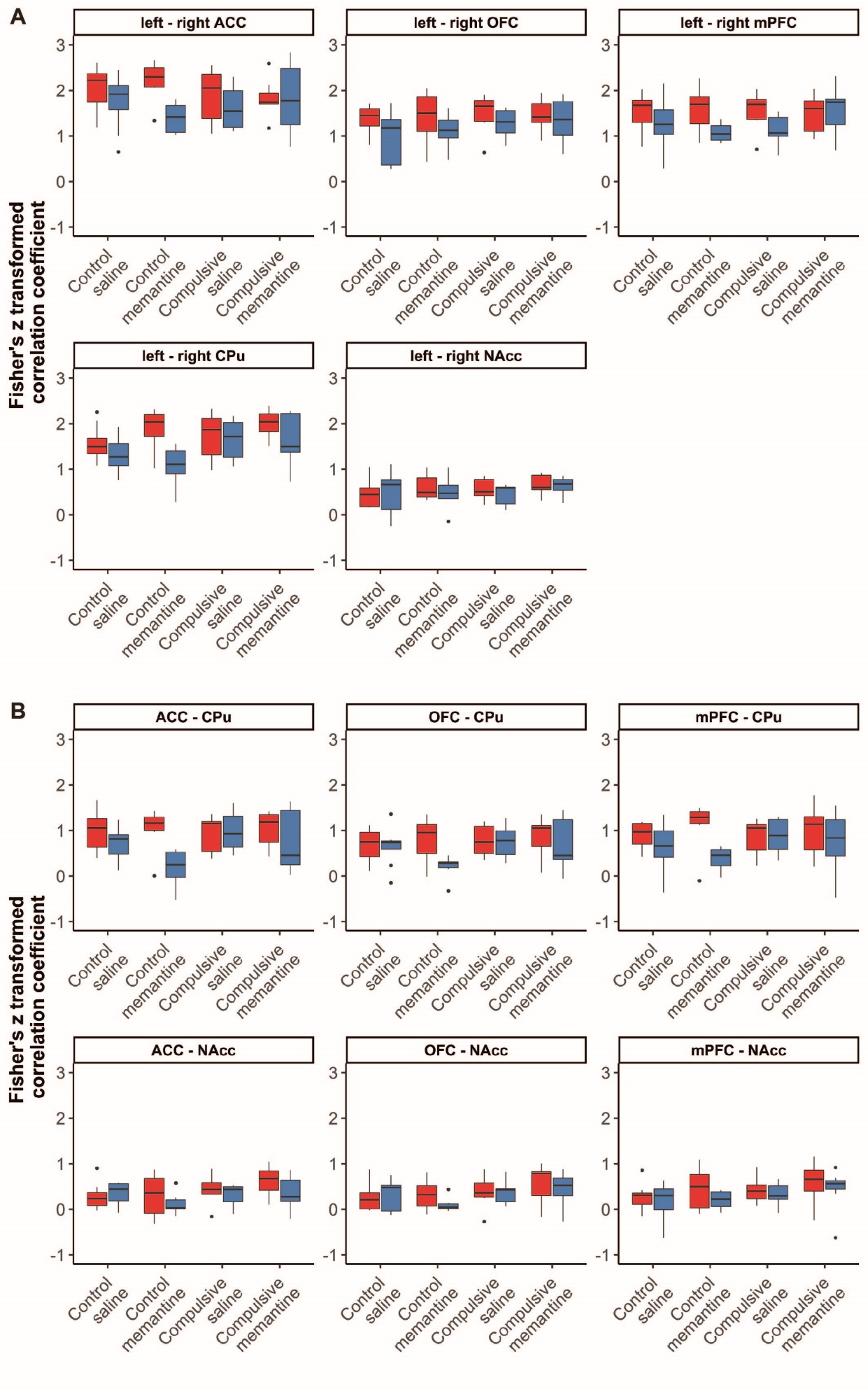
Supplementary Figure S3: Functional connectivity between individual brain regions in the frontostriatal system before and after saline/memantine treatment in control and compulsive rats.** Bar graphs of interhemispheric (A) and intrahemispheric (B) functional connectivity (Fisher’s Z-transformed correlation coefficient) between individual sub-regions within the frontostriatal system before (red) and after (blue) seven days of daily saline/memantine treatment (Control + saline: n=8; Control + memantine: n=6; Compulsive + saline: n=7; Compulsive + memantine: n=7). Error bars represent 1.5 times the interquartile range, and dots represent values that exceeded 1.5 times the interquartile range.


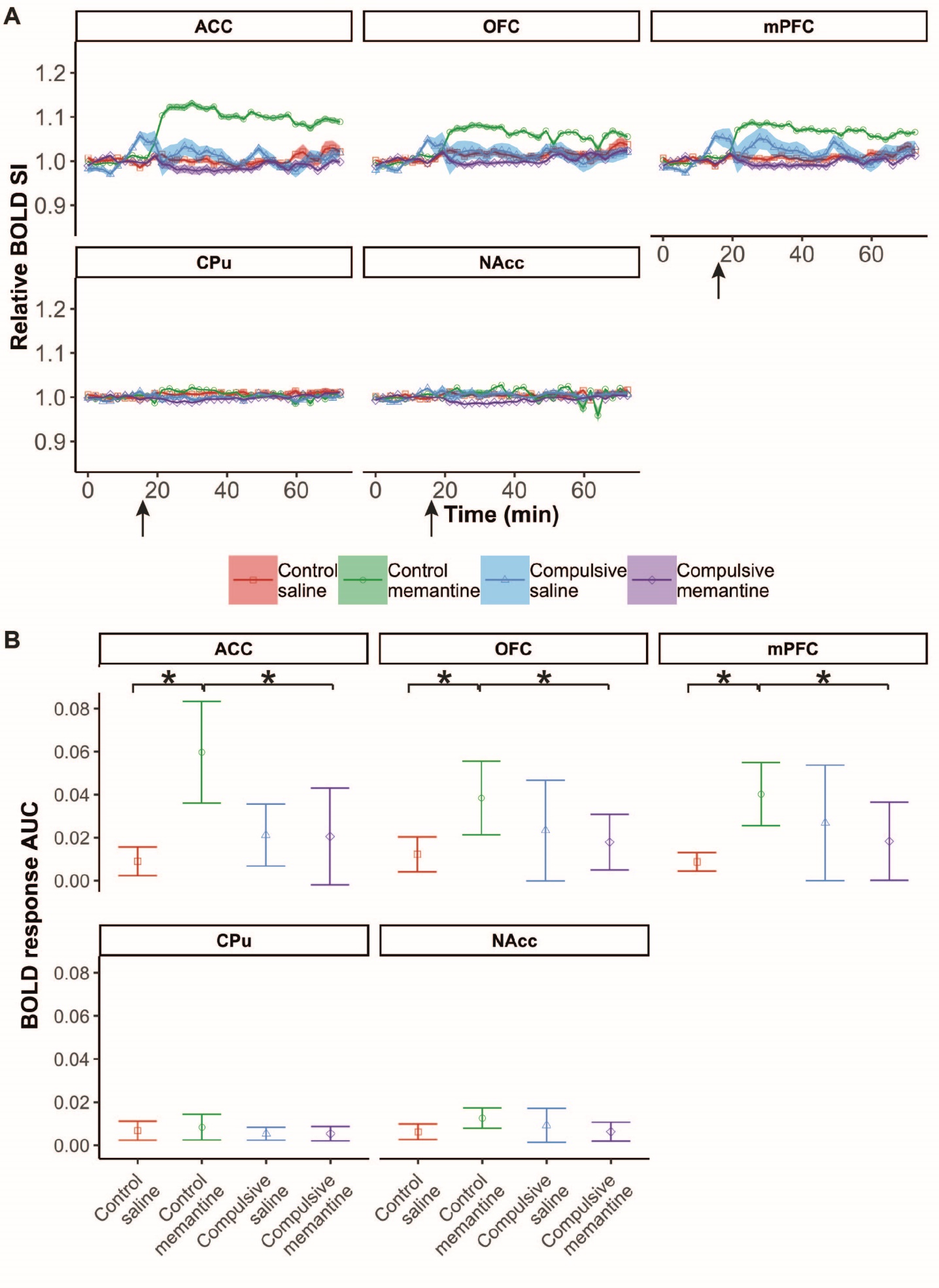


**Supplementary Figure S4: Brain activation directly after memantine/saline injection in control and compulsive rats in sub-regions of the frontostriatal system.** The normalized BOLD signal intensity (SI) time-course is shown as averaged time series for the regions-of-interest, with the arrow indicating the time of memantine/saline injection (A). BOLD responses to memantine or saline injection quantified as AUC (relative positive BOLD SI change per second) (B). * corrected p<0.05. Shades in A represent the standard error. Error bars in B represent the standard deviation.

**Supplementary Table 1: Statistical results of mixed design ANOVA for compulsive behavioral measures and body weight.**

| Variable | Effect | DFn | DFd | SSn | SSd | F | p | p.fdr |
| --- | --- | --- | --- | --- | --- | --- | --- | --- |
| stops before returning to homebase | (Intercept) | 1 | 24 | 4593.89896462974 | 300.550088329912 | 366.839270498197 | 4.77027054230984e-16 | 1.71729739523154e-15 |
| recurrence time of checking | (Intercept) | 1 | 24 | 105488.914921348 | 38471.2926789235 | 65.8083932672054 | 2.46407735837327e-08 | 4.43533924507189e-08 |
| mean velocity | (Intercept) | 1 | 24 | 7234.92892646655 | 895.499541722392 | 193.901041982918 | 5.4203735715062e-13 | 1.39381034695874e-12 |
| frequency of checking | (Intercept) | 1 | 24 | 109209.446428571 | 26002.3898809524 | 100.799454445751 | 4.54752271086301e-10 | 1.02319260994418e-09 |
| length of checks | (Intercept) | 1 | 24 | 6698.17758861796 | 12313.3879796006 | 13.0554046045778 | 0.00139105887120162 | 0.00147288586362524 |
| immobility time | (Intercept) | 1 | 24 | 1521445.84305301 | 489991.264164173 | 74.5211249746647 | 7.9962204259738e-09 | 1.59924408519476e-08 |
| total distance | (Intercept) | 1 | 24 | 8937153076.22364 | 1056976691.59585 | 202.929426481035 | 3.32257283213968e-13 | 9.96771849641904e-13 |
| entropy | (Intercept) | 1 | 24 | 260.481040327234 | 0.094554543195622 | 66115.7545324915 | 8.40172666219662e-43 | 1.51231079919539e-41 |
| body weight | (Intercept) | 1 | 24 | 6921738.28571428 | 49018.8095238095 | 3388.93825596588 | 2.36439339448028e-27 | 2.12795405503225e-26 |
| Behavioral acts | (Intercept) | 1 | 24 | 118.947525795625 | 103.665148923053 | 27.5380940340323 | 2.22243398215758e-05 | 3.63671015262149e-05 |
| clockwise | (Intercept) | 1 | 24 | 10.3157566197414 | 10.0508581135213 | 24.6325394386703 | 4.56425005753957e-05 | 6.31973084890094e-05 |
| counter clockwise | (Intercept) | 1 | 24 | 10.494404176751 | 12.7865675425989 | 19.6976787869711 | 0.000173322097968785 | 0.000207986517562542 |
| forelegs | (Intercept) | 1 | 24 | 1.00976967019887 | 1.16020174039613 | 20.8881535348314 | 0.000123833487906938 | 0.000159214484451777 |
| grooming time | (Intercept) | 1 | 24 | 9.74803714688218 | 75.7055948056892 | 3.09029857206262 | 0.0915094528710615 | 0.0915094528710615 |
| headdip | (Intercept) | 1 | 24 | 0.0754877990925872 | 0.107249080475386 | 16.8925194527695 | 0.00039884331879227 | 0.000448698733641304 |
| sniffing | (Intercept) | 1 | 24 | 8.26601820322341 | 7.84827890978986 | 25.2774447949218 | 3.87544099408055e-05 | 5.81316149112082e-05 |
| Enter | (Intercept) | 1 | 24 | 77144.8246351406 | 3718.75619227353 | 497.875013987255 | 1.47390661781809e-17 | 8.84343970690854e-17 |
| Leave | (Intercept) | 1 | 24 | 75259.8823827308 | 4049.1625069406 | 446.076731691923 | 5.17972618349172e-17 | 2.33087678257127e-16 |
| stops before returning to homebase | group:treatment:time | 1 | 24 | 0.00253523589614202 | 55.4758379880384 | 0.00109679571709269 | 0.973854457910809 | 0.973854457910809 |
| recurrence time of checking | group:treatment:time | 1 | 24 | 675.703824292185 | 14271.2084929728 | 1.13633626689693 | 0.297034746686838 | 0.536483347121 |
| mean velocity | group:treatment:time | 1 | 24 | 8.70494247286051 | 410.691953063409 | 0.508699081611653 | 0.482580770298599 | 0.658208337892797 |
| frequency of checking | group:treatment:time | 1 | 24 | 150.806608983799 | 8166.79464285714 | 0.443179824385171 | 0.511939818361064 | 0.658208337892797 |
| length of checks | group:treatment:time | 1 | 24 | 1105.02888631913 | 6020.76024968003 | 4.40487449621807 | 0.0465386599129701 | 0.400712259163058 |
| immobility time | group:treatment:time | 1 | 24 | 13023.974807217 | 135349.820294175 | 2.30938906822222 | 0.141660129675306 | 0.400712259163058 |
| total distance | group:treatment:time | 1 | 24 | 25729186.549388 | 436592572.731021 | 1.41436322043377 | 0.245966976332672 | 0.536483347121 |
| entropy | group:treatment:time | 1 | 24 | 0.000956953291605665 | 0.0446932549004267 | 0.513877967709098 | 0.4803836361924 | 0.658208337892797 |
| body weight | group:treatment:time | 1 | 24 | 2.0741286205205 | 1257.52380952381 | 0.0395850054810031 | 0.843970098025309 | 0.949466360278473 |
| Behavioral acts | group:treatment:time | 1 | 24 | 5.2989256834011 | 46.6050156676492 | 2.72876673421879 | 0.111580721772575 | 0.400712259163058 |
| clockwise | group:treatment:time | 1 | 24 | 0.509645157963821 | 5.69715157625052 | 2.14694722922953 | 0.155832545230078 | 0.400712259163058 |
| counter clockwise | group:treatment:time | 1 | 24 | 0.58747935968072 | 2.674315114944 | 5.27219270217994 | 0.0307042238770857 | 0.400712259163058 |
| forelegs | group:treatment:time | 1 | 24 | 0.0439197019650361 | 0.93158952157493 | 1.131477783669 | 0.298046303956111 | 0.536483347121 |
| grooming time | group:treatment:time | 1 | 24 | 4.99158843078443 | 38.9205762751357 | 3.07801512217996 | 0.0921187650690326 | 0.400712259163058 |
| headdip | group:treatment:time | 1 | 24 | 0.0182459497347235 | 0.198905572131877 | 2.20156121791816 | 0.150886233452505 | 0.400712259163058 |
| sniffing | group:treatment:time | 1 | 24 | 0.117660221306601 | 5.35234155451813 | 0.527590640955021 | 0.474646603435031 | 0.658208337892797 |
| Enter | group:treatment:time | 1 | 24 | 0.581542013097947 | 2702.43198786883 | 0.00516461038686764 | 0.943304706127031 | 0.973854457910809 |
| Leave | group:treatment:time | 1 | 24 | 32.0206804238025 | 3614.24921761555 | 0.212629590241224 | 0.648865812792617 | 0.77863897535114 |
| stops before returning to homebase | treatment:time | 1 | 24 | 1.35554523710083 | 55.4758379880384 | 0.586437030431782 | 0.451266476750839 | 0.613669025554274 |
| recurrence time of checking | treatment:time | 1 | 24 | 319.242184554827 | 14271.2084929728 | 0.5368720128424 | 0.470828190725166 | 0.613669025554274 |
| mean velocity | treatment:time | 1 | 24 | 8.54877279488287 | 410.691953063409 | 0.499572844188431 | 0.486494221234698 | 0.613669025554274 |
| frequency of checking | treatment:time | 1 | 24 | 219.434462444772 | 8166.79464285714 | 0.64485851903729 | 0.429838356231103 | 0.613669025554274 |
| length of checks | treatment:time | 1 | 24 | 776.046602191475 | 6020.76024968003 | 3.09348282944587 | 0.0913522600387275 | 0.428351201697596 |
| immobility time | treatment:time | 1 | 24 | 12199.4478245008 | 135349.820294175 | 2.16318534558573 | 0.154341851180781 | 0.463025553542343 |
| total distance | treatment:time | 1 | 24 | 29685434.3549835 | 436592572.731021 | 1.63184275001063 | 0.213668070937389 | 0.549432182410429 |
| entropy | treatment:time | 1 | 24 | 0.00206065623345985 | 0.0446932549004267 | 1.1065595851817 | 0.303307776810943 | 0.606615553621886 |
| body weight | treatment:time | 1 | 24 | 1535.25920471281 | 1257.52380952381 | 29.3006149339313 | 1.46615547803111e-05 | 0.0002639079860456 |
| Behavioral acts | treatment:time | 1 | 24 | 0.610003110883363 | 46.6050156676492 | 0.314130881654506 | 0.580351305433765 | 0.652895218612986 |
| clockwise | treatment:time | 1 | 24 | 0.105479073295137 | 5.69715157625052 | 0.444344463229 | 0.511390854628562 | 0.613669025554274 |
| counter clockwise | treatment:time | 1 | 24 | 0.0663659791714898 | 2.674315114944 | 0.595585572999727 | 0.447800049240539 | 0.613669025554274 |
| forelegs | treatment:time | 1 | 24 | 0.00744617437235699 | 0.93158952157493 | 0.191831467398266 | 0.665313404000641 | 0.704449486588914 |
| grooming time | treatment:time | 1 | 24 | 4.88971927913578 | 38.9205762751357 | 3.01519848703343 | 0.0953089634386427 | 0.428351201697596 |
| headdip | treatment:time | 1 | 24 | 0.0114709894089719 | 0.198905572131877 | 1.38409267706586 | 0.250942789312521 | 0.564621275953172 |
| sniffing | treatment:time | 1 | 24 | 0.00616956391273601 | 5.35234155451813 | 0.0276644403944425 | 0.869293471813513 | 0.869293471813513 |
| Enter | treatment:time | 1 | 24 | 418.538233611979 | 2702.43198786883 | 3.71699182505941 | 0.0657747953663976 | 0.428351201697596 |
| Leave | treatment:time | 1 | 24 | 393.669699620573 | 3614.24921761555 | 2.61411768309851 | 0.118986444915999 | 0.428351201697596 |
| stops before returning to homebase | group:time | 1 | 24 | 4.90424797016694 | 55.4758379880384 | 2.12167955551001 | 0.15818668812306 | 0.412973268756851 |
| recurrence time of checking | group:time | 1 | 24 | 68.1471710840472 | 14271.2084929728 | 0.114603616562849 | 0.737906479325203 | 0.830144789240853 |
| mean velocity | group:time | 1 | 24 | 140.621529798417 | 410.691953063409 | 8.21763535902768 | 0.00849997991480318 | 0.152999638466457 |
| frequency of checking | group:time | 1 | 24 | 948.326953287262 | 8166.79464285714 | 2.78687635409083 | 0.108033492063593 | 0.412973268756851 |
| length of checks | group:time | 1 | 24 | 84.5706526648692 | 6020.76024968003 | 0.3371161746666 | 0.56691110152186 | 0.796436254519333 |
| immobility time | group:time | 1 | 24 | 10576.1242209362 | 135349.820294175 | 1.87534036432993 | 0.183543675003045 | 0.412973268756851 |
| total distance | group:time | 1 | 24 | 65962556.0331233 | 436592572.731021 | 3.62603819595962 | 0.0689376074723548 | 0.412973268756851 |
| entropy | group:time | 1 | 24 | 0.000232195037056153 | 0.0446932549004267 | 0.124687291220189 | 0.727087975730649 | 0.830144789240853 |
| body weight | group:time | 1 | 24 | 0.45334390695214 | 1257.52380952381 | 0.00865212545834135 | 0.926662352055348 | 0.955498013571912 |
| Behavioral acts | group:time | 1 | 24 | 1.6952977746563 | 46.6050156676492 | 0.873020768449053 | 0.359426733265445 | 0.71885346653089 |
| clockwise | group:time | 1 | 24 | 0.483840169116718 | 5.69715157625052 | 2.03824032121743 | 0.166270504729259 | 0.412973268756851 |
| counter clockwise | group:time | 1 | 24 | 0.217096364953211 | 2.674315114944 | 1.94827929205574 | 0.175552485618838 | 0.412973268756851 |
| forelegs | group:time | 1 | 24 | 0.000123428825245797 | 0.93158952157493 | 0.00317982516687299 | 0.955498013571912 | 0.955498013571912 |
| grooming time | group:time | 1 | 24 | 3.95131580012017 | 38.9205762751357 | 2.43654098368186 | 0.131628149906885 | 0.412973268756851 |
| headdip | group:time | 1 | 24 | 0.0040975159369596 | 0.198905572131877 | 0.494407378501339 | 0.488733234215308 | 0.796436254519333 |
| sniffing | group:time | 1 | 24 | 0.071990345796617 | 5.35234155451813 | 0.322806061892729 | 0.575203961597296 | 0.796436254519333 |
| Enter | group:time | 1 | 24 | 51.7620080941158 | 2702.43198786883 | 0.459692676757598 | 0.504252544730121 | 0.796436254519333 |
| Leave | group:time | 1 | 24 | 26.9317406948645 | 3614.24921761555 | 0.178837080057024 | 0.676139725117015 | 0.830144789240853 |
| stops before returning to homebase | group:treatment | 1 | 24 | 2.35257281494546 | 300.550088329912 | 0.187861357394489 | 0.668573438854672 | 0.707901288199065 |
| recurrence time of checking | group:treatment | 1 | 24 | 2182.88289678726 | 38471.2926789235 | 1.36177356867438 | 0.254694729377459 | 0.492782670156742 |
| mean velocity | group:treatment | 1 | 24 | 16.186796807953 | 895.499541722392 | 0.433817221886758 | 0.516391432356866 | 0.580940361401474 |
| frequency of checking | group:treatment | 1 | 24 | 595.361591801668 | 26002.3898809524 | 0.549514035773572 | 0.465708543215564 | 0.572256392299055 |
| length of checks | group:treatment | 1 | 24 | 2238.55217336794 | 12313.3879796006 | 4.36315758504777 | 0.047504693027458 | 0.361389542189638 |
| immobility time | group:treatment | 1 | 24 | 22799.5323224825 | 489991.264164173 | 1.1167316965803 | 0.301144965095787 | 0.492782670156742 |
| total distance | group:treatment | 1 | 24 | 29328541.0793598 | 1056976691.59585 | 0.665941823979007 | 0.422496004057323 | 0.572256392299055 |
| entropy | group:treatment | 1 | 24 | 0.000178346732265846 | 0.094554543195622 | 0.0452682803990162 | 0.833308314498212 | 0.833308314498212 |
| body weight | group:treatment | 1 | 24 | 1066.60579283259 | 49018.8095238095 | 0.522218700875395 | 0.476880326915879 | 0.572256392299055 |
| Behavioral acts | group:treatment | 1 | 24 | 12.6129564243794 | 103.665148923053 | 2.920084110522 | 0.100385983941566 | 0.361389542189638 |
| clockwise | group:treatment | 1 | 24 | 1.25752101780917 | 10.0508581135213 | 3.0027788758473 | 0.0959547261828826 | 0.361389542189638 |
| counter clockwise | group:treatment | 1 | 24 | 1.26148445946063 | 12.7865675425989 | 2.36776812277344 | 0.136945572926867 | 0.410836718780601 |
| forelegs | group:treatment | 1 | 24 | 0.0573354466218039 | 1.16020174039613 | 1.18604434988476 | 0.286944089583531 | 0.492782670156742 |
| grooming time | group:treatment | 1 | 24 | 9.3950491081245 | 75.7055948056892 | 2.97839517903165 | 0.0972373528112949 | 0.361389542189638 |
| headdip | group:treatment | 1 | 24 | 0.0196693497405977 | 0.107249080475386 | 4.40157054663688 | 0.0466143573112195 | 0.361389542189638 |
| sniffing | group:treatment | 1 | 24 | 0.536684712579503 | 7.84827890978986 | 1.64117932733522 | 0.212404728644375 | 0.477910639449844 |
| Enter | group:treatment | 1 | 24 | 265.408650553793 | 3718.75619227353 | 1.71288659001781 | 0.20300564529069 | 0.477910639449844 |
| Leave | group:treatment | 1 | 24 | 88.2606649330982 | 4049.1625069406 | 0.523134340684892 | 0.476498349350243 | 0.572256392299055 |
| stops before returning to homebase | group | 1 | 24 | 118.050254637649 | 300.550088329912 | 9.42673524751583 | 0.00524802746321042 | 0.0134949277625411 |
| recurrence time of checking | group | 1 | 24 | 44416.6957964952 | 38471.2926789235 | 27.7089909094709 | 2.13319317136942e-05 | 9.59936927116239e-05 |
| mean velocity | group | 1 | 24 | 1781.1398610059 | 895.499541722392 | 47.7357660975705 | 3.81425457017897e-07 | 3.43282911316107e-06 |
| frequency of checking | group | 1 | 24 | 19865.6023733998 | 26002.3898809524 | 18.3357937152865 | 0.00025780570591151 | 0.000928100541281436 |
| length of checks | group | 1 | 24 | 1603.14365079809 | 12313.3879796006 | 3.12468409855157 | 0.0898283593952291 | 0.161691046911412 |
| immobility time | group | 1 | 24 | 1110071.18286495 | 489991.264164173 | 54.3718027997995 | 1.2948400858814e-07 | 2.33071215458652e-06 |
| total distance | group | 1 | 24 | 328005618.146893 | 1056976691.59585 | 7.44778470340714 | 0.0116989648535487 | 0.0263226709204846 |
| entropy | group | 1 | 24 | 0.12126915461783 | 0.094554543195622 | 30.7807495278838 | 1.04534034500635e-05 | 6.2720420700381e-05 |
| body weight | group | 1 | 24 | 2152.24622181941 | 49018.8095238095 | 1.05375691138677 | 0.314879047845348 | 0.404844490086876 |
| Behavioral acts | group | 1 | 24 | 4.82266227283598 | 103.665148923053 | 1.11651693698888 | 0.301190412716461 | 0.404844490086876 |
| clockwise | group | 1 | 24 | 0.0277757209705436 | 10.0508581135213 | 0.0663244168571293 | 0.798960912692359 | 0.829654559030672 |
| counter clockwise | group | 1 | 24 | 0.814738120324798 | 12.7865675425989 | 1.52923877519524 | 0.228191964925317 | 0.373405033514155 |
| forelegs | group | 1 | 24 | 0.0574264232059833 | 1.16020174039613 | 1.18792629674304 | 0.286571088246874 | 0.404844490086876 |
| grooming time | group | 1 | 24 | 11.0983344973135 | 75.7055948056892 | 3.51836649086743 | 0.0729081867181923 | 0.145816373436385 |
| headdip | group | 1 | 24 | 0.0652412866521022 | 0.107249080475386 | 14.5995739330354 | 0.000827371948917678 | 0.00248211584675303 |
| sniffing | group | 1 | 24 | 0.15642360689192 | 7.84827890978986 | 0.478342654301337 | 0.495810743603883 | 0.59497289232466 |
| Enter | group | 1 | 24 | 7.80486955413608 | 3718.75619227353 | 0.050370838961816 | 0.824318994309843 | 0.829654559030672 |
| Leave | group | 1 | 24 | 7.98153347891881 | 4049.1625069406 | 0.0473077588675947 | 0.829654559030672 | 0.829654559030672 |
| stops before returning to homebase | treatment | 1 | 24 | 11.8547036002856 | 300.550088329912 | 0.94664050171413 | 0.340280781025493 | 0.702161826383604 |
| recurrence time of checking | treatment | 1 | 24 | 912.918318585128 | 38471.2926789235 | 0.569516595891421 | 0.457792901100542 | 0.824027221980976 |
| mean velocity | treatment | 1 | 24 | 0.148204039266322 | 895.499541722392 | 0.00397196958420596 | 0.950269704308204 | 0.950269704308204 |
| frequency of checking | treatment | 1 | 24 | 144.284241531664 | 26002.3898809524 | 0.133173212639834 | 0.71836050976463 | 0.933924565344592 |
| length of checks | treatment | 1 | 24 | 1164.71575035945 | 12313.3879796006 | 2.27014515054154 | 0.144937964268666 | 0.63272279649105 |
| immobility time | treatment | 1 | 24 | 33734.5474403764 | 489991.264164173 | 1.65233382262457 | 0.21090759883035 | 0.63272279649105 |
| total distance | treatment | 1 | 24 | 6276479.99491761 | 1056976691.59585 | 0.142515460440844 | 0.709110916367021 | 0.933924565344592 |
| entropy | treatment | 1 | 24 | 0.000218943123265383 | 0.094554543195622 | 0.0555725275674801 | 0.815635847534653 | 0.950269704308204 |
| body weight | treatment | 1 | 24 | 57.1561119293065 | 49018.8095238095 | 0.0279840881414525 | 0.868547780583683 | 0.950269704308204 |
| Behavioral acts | treatment | 1 | 24 | 0.558839321340772 | 103.665148923053 | 0.129379486273963 | 0.722222040744435 | 0.933924565344592 |
| clockwise | treatment | 1 | 24 | 0.0524983008144628 | 10.0508581135213 | 0.125358372918637 | 0.726385773045794 | 0.933924565344592 |
| counter clockwise | treatment | 1 | 24 | 0.00273173624577638 | 12.7865675425989 | 0.00512738619494341 | 0.943509027956856 | 0.950269704308204 |
| forelegs | treatment | 1 | 24 | 0.095832324930797 | 1.16020174039613 | 1.98239299102745 | 0.171960550048596 | 0.63272279649105 |
| grooming time | treatment | 1 | 24 | 9.20331341204033 | 75.7055948056892 | 2.91761160394937 | 0.100522082624605 | 0.63272279649105 |
| headdip | treatment | 1 | 24 | 0.012593231591597 | 0.107249080475386 | 2.81808997204124 | 0.106182427965227 | 0.63272279649105 |
| sniffing | treatment | 1 | 24 | 0.295753561960396 | 7.84827890978986 | 0.904413001708622 | 0.351080913191802 | 0.702161826383604 |
| Enter | treatment | 1 | 24 | 301.061432715708 | 3718.75619227353 | 1.94298147326501 | 0.17611845435243 | 0.63272279649105 |
| Leave | treatment | 1 | 24 | 171.739994341266 | 4049.1625069406 | 1.01792898090046 | 0.323075175556107 | 0.702161826383604 |
| stops before returning to homebase | time | 1 | 24 | 3.48095599344425 | 55.4758379880384 | 1.5059338781088 | 0.231662628287571 | 0.470048190663238 |
| recurrence time of checking | time | 1 | 24 | 368.66584279608 | 14271.2084929728 | 0.619988155275198 | 0.438759078634646 | 0.564118815387402 |
| mean velocity | time | 1 | 24 | 271.023932809069 | 410.691953063409 | 15.8380857937418 | 0.00055461982216955 | 0.00499157839952595 |
| frequency of checking | time | 1 | 24 | 3225.44642857142 | 8166.79464285714 | 9.47871443705509 | 0.00514290513421127 | 0.0231430731039507 |
| length of checks | time | 1 | 24 | 853.747351203448 | 6020.76024968003 | 3.40321414226246 | 0.0774465633318045 | 0.232339689995413 |
| immobility time | time | 1 | 24 | 30332.9576614471 | 135349.820294175 | 5.37858847756492 | 0.0292172919518007 | 0.105182251026483 |
| total distance | time | 1 | 24 | 264405579.441833 | 436592572.731021 | 14.5346813091883 | 0.000845229865409195 | 0.00507137919245517 |
| entropy | time | 1 | 24 | 0.000283911048236898 | 0.0446932549004267 | 0.152458467678542 | 0.69964117355789 | 0.740796536708354 |
| body weight | time | 1 | 24 | 2972.57142857141 | 1257.52380952381 | 56.7318994244165 | 9.01765458827629e-08 | 1.62317782588973e-06 |
| Behavioral acts | time | 1 | 24 | 2.30014182684694 | 46.6050156676492 | 1.18449491011857 | 0.287251672071979 | 0.470048190663238 |
| clockwise | time | 1 | 24 | 0.295563036460106 | 5.69715157625052 | 1.24509814775036 | 0.275538965900999 | 0.470048190663238 |
| counter clockwise | time | 1 | 24 | 0.0285375255817164 | 2.674315114944 | 0.256103183253906 | 0.617427173196624 | 0.694605569846202 |
| forelegs | time | 1 | 24 | 0.0187919705679197 | 0.93158952157493 | 0.484126627860311 | 0.493242346517271 | 0.591890815820725 |
| grooming time | time | 1 | 24 | 3.36409989728111 | 38.9205762751357 | 2.07443993028248 | 0.162703675254129 | 0.418380879224903 |
| headdip | time | 1 | 24 | 0.00692443785065448 | 0.198905572131877 | 0.835504539337508 | 0.369777882051448 | 0.546629195467677 |
| sniffing | time | 1 | 24 | 0.269485616729527 | 5.35234155451813 | 1.20837856396684 | 0.282558599935699 | 0.470048190663238 |
| Enter | time | 1 | 24 | 84.5467568410236 | 2702.43198786883 | 0.750850409295501 | 0.394787752282211 | 0.546629195467677 |
| Leave | time | 1 | 24 | 0.427773334018866 | 3614.24921761555 | 0.00284057888604207 | 0.957936368016316 | 0.957936368016316 |

DFn = Degrees of freedom in the numerator, DFd = degrees of freedom in the denominator, SSn = sum of squares in the numerator, SSd = sum of squares in the denominator, F= F-value, p = p-value, p.fdr = fdr-corrected p-value.

**Supplementary Table 2: Statistical results of Wilcoxon Signed Rank tests for compulsive behavioral measures and body weight.**

| variable | group | Statistic | p.value | fdr |
| --- | --- | --- | --- | --- |
| stops before returning to homebase | Compulsive + saline | 10 | 0.578125 | 0.88445782743985 |
| stops before returning to homebase | Compulsive + memantine | 1 | 0.03125 | 0.26953125 |
| stops before returning to homebase | Control + saline | 24 | 0.4609375 | 0.80859375 |
| stops before returning to homebase | Control + memantine | 15 | 0.4375 | 0.80859375 |
| recurrence time of checking | Compulsive + saline | 24 | 0.109375 | 0.559027777777778 |
| recurrence time of checking | Compulsive + memantine | 24 | 0.109375 | 0.559027777777778 |
| recurrence time of checking | Control + saline | 24 | 0.4609375 | 0.80859375 |
| recurrence time of checking | Control + memantine | 11 | 1 | 1 |
| mean velocity | Compulsive + saline | 1 | 0.03125 | 0.26953125 |
| mean velocity | Compulsive + memantine | 0 | 0.015625 | 0.26953125 |
| mean velocity | Control + saline | 21 | 0.7421875 | 1 |
| mean velocity | Control + memantine | 4 | 0.21875 | 0.559027777777778 |
| frequency of checking | Compulsive + saline | 6 | 0.21875 | 0.559027777777778 |
| frequency of checking | Compulsive + memantine | 4.5 | 0.127508330490583 | 0.559027777777778 |
| frequency of checking | Control + saline | 17.5 | 1 | 1 |
| frequency of checking | Control + memantine | 3.5 | 0.171772777595712 | 0.559027777777778 |
| length of checks | Compulsive + saline | 19 | 0.46875 | 0.80859375 |
| length of checks | Compulsive + memantine | 14 | 1 | 1 |
| length of checks | Control + saline | 12 | 0.4609375 | 0.80859375 |
| length of checks | Control + memantine | 17 | 0.21875 | 0.559027777777778 |
| immobility time | Compulsive + saline | 24 | 0.109375 | 0.559027777777778 |
| immobility time | Compulsive + memantine | 27 | 0.03125 | 0.26953125 |
| immobility time | Control + saline | 20 | 0.84375 | 1 |
| immobility time | Control + memantine | 18 | 0.15625 | 0.559027777777778 |
| total distance | Compulsive + saline | 1 | 0.03125 | 0.26953125 |
| total distance | Compulsive + memantine | 0 | 0.015625 | 0.26953125 |
| total distance | Control + saline | 21 | 0.7421875 | 1 |
| total distance | Control + memantine | 4 | 0.21875 | 0.559027777777778 |
| entropy | Compulsive + saline | 16 | 0.8125 | 1 |
| entropy | Compulsive + memantine | 6 | 0.21875 | 0.559027777777778 |
| entropy | Control + saline | 23 | 0.546875 | 0.88445782743985 |
| entropy | Control + memantine | 12 | 0.84375 | 1 |
| body weight | Compulsive + saline | 0 | 0.015625 | 0.26953125 |
| body weight | Compulsive + memantine | 3 | 0.073834315161456 | 0.559027777777778 |
| body weight | Control + saline | 0 | 0.0140290608983004 | 0.26953125 |
| body weight | Control + memantine | 7 | 1 | 1 |
| Behavioral acts | Compulsive + saline | 17 | 0.6875 | 0.98828125 |
| Behavioral acts | Compulsive + memantine | 11 | 1 | 1 |
| Behavioral acts | Control + saline | 16 | 0.84375 | 1 |
| Behavioral acts | Control + memantine | 18 | 0.15625 | 0.559027777777778 |
| clockwise | Compulsive + saline | 10 | 0.589638551626567 | 0.88445782743985 |
| clockwise | Compulsive + memantine | 10 | 0.589638551626567 | 0.88445782743985 |
| clockwise | Control + saline | 12 | 0.4609375 | 0.80859375 |
| clockwise | Control + memantine | 16 | 0.3125 | 0.695564516129032 |
| counter clockwise | Compulsive + saline | 12 | 0.280712665268496 | 0.667902548397456 |
| counter clockwise | Compulsive + memantine | 11 | 1 | 1 |
| counter clockwise | Control + saline | 4 | 0.108319380730004 | 0.559027777777778 |
| counter clockwise | Control + memantine | 16 | 0.3125 | 0.695564516129032 |
| forelegs | Compulsive + saline | 4 | 0.789268026134281 | 1 |
| forelegs | Compulsive + memantine | 2 | 1 | 1 |
| forelegs | Control + saline | 9 | 0.833935414088552 | 1 |
| forelegs | Control + memantine | 9 | 0.201242620957724 | 0.559027777777778 |
| grooming time | Compulsive + saline | 0 | NA | NA |
| grooming time | Compulsive + memantine | 0 | NA | NA |
| grooming time | Control + saline | 3 | 1 | 1 |
| grooming time | Control + memantine | 6 | 0.18144920772142 | 0.559027777777778 |
| headdip | Compulsive + saline | 1 | 1 | 1 |
| headdip | Compulsive + memantine | 0 | NA | NA |
| headdip | Control + saline | 4 | 0.855132140584706 | 1 |
| headdip | Control + memantine | 12 | 0.280712665268496 | 0.667902548397456 |
| sniffing | Compulsive + saline | 14.5 | 0.461838018150638 | 0.80859375 |
| sniffing | Compulsive + memantine | 9 | 0.787406490666269 | 1 |
| sniffing | Control + saline | 10 | 1 | 1 |
| sniffing | Control + memantine | 9 | 0.201242620957724 | 0.559027777777778 |
| Enter | Compulsive + saline | 6 | 0.201642922559149 | 0.559027777777778 |
| Enter | Compulsive + memantine | 16.5 | 0.734402143294063 | 1 |
| Enter | Control + saline | 11 | 0.3828125 | 0.80859375 |
| Enter | Control + memantine | 14 | 0.5625 | 0.88445782743985 |
| Leave | Compulsive + saline | 6 | 0.401678166469773 | 0.80859375 |
| Leave | Compulsive + memantine | 13 | 0.177529852412153 | 0.559027777777778 |
| Leave | Control + saline | 14.5 | 0.674047352705026 | 0.98828125 |
| Leave | Control + memantine | 10 | 0.589638551626567 | 0.88445782743985 |

Statistic = V, p = p-value, p.fdr = fdr-corrected p-value.

**Supplementary Table 3: Statistical results of mixed design ANOVA for functional connectivity measured with resting-state fMRI.**

| variable | Effect | DFn | DFd | SSn | SSd | F | p | p.fdr |
| --- | --- | --- | --- | --- | --- | --- | --- | --- |
| frontal cortex - striatum | (Intercept) | 1 | 24 | 44.0640868693475 | 6.61599363445805 | 159.845692619226 | 4.21023036750401e-12 | 4.21023036750401e-12 |
| left frontal cortex - right frontal cortex | (Intercept) | 1 | 24 | 200.321438755249 | 10.5211433278535 | 456.957421860998 | 3.93377343724043e-17 | 5.90066015586065e-17 |
| left striatum - right striatum | (Intercept) | 1 | 24 | 146.161626023178 | 6.96758534660439 | 503.456915137153 | 1.29694704388702e-17 | 3.89084113166105e-17 |
| frontal cortex - striatum | group:treatment:time | 1 | 24 | 0.0593105343839428 | 5.48057524345285 | 0.259726901280135 | 0.614962389053891 | 0.614962389053891 |
| left frontal cortex - right frontal cortex | group:treatment:time | 1 | 24 | 0.307188645483222 | 6.44868115294685 | 1.14326128346853 | 0.295600864850692 | 0.614962389053891 |
| left striatum - right striatum | group:treatment:time | 1 | 24 | 0.103087440956064 | 3.54003071720201 | 0.698891840379571 | 0.411402610895249 | 0.614962389053891 |
| frontal cortex - striatum | treatment:time | 1 | 24 | 0.646706494526681 | 5.48057524345285 | 2.8319939384432 | 0.105369782990954 | 0.158054674486431 |
| left frontal cortex - right frontal cortex | treatment:time | 1 | 24 | 0.102885219817529 | 6.44868115294685 | 0.38290701882389 | 0.541880886157711 | 0.541880886157711 |
| left striatum - right striatum | treatment:time | 1 | 24 | 0.618404649132999 | 3.54003071720201 | 4.19253751304244 | 0.0516979675692811 | 0.155093902707843 |
| frontal cortex - striatum | group:time | 1 | 24 | 0.623708409052957 | 5.48057524345285 | 2.7312829679974 | 0.111424333671155 | 0.199231921656906 |
| left frontal cortex - right frontal cortex | group:time | 1 | 24 | 0.468294056830772 | 6.44868115294685 | 1.74284587768813 | 0.199231921656906 | 0.199231921656906 |
| left striatum - right striatum | group:time | 1 | 24 | 0.304216585369743 | 3.54003071720201 | 2.06246742814836 | 0.16387304225413 | 0.199231921656906 |
| frontal cortex - striatum | group:treatment | 1 | 24 | 0.143917897619763 | 6.61599363445805 | 0.522072682308022 | 0.476941288939323 | 0.793727582057187 |
| left frontal cortex - right frontal cortex | group:treatment | 1 | 24 | 0.0306465012012966 | 10.5211433278535 | 0.0699083745854811 | 0.793727582057187 | 0.793727582057187 |
| left striatum - right striatum | group:treatment | 1 | 24 | 0.0595747578403514 | 6.96758534660439 | 0.205206555362144 | 0.654619538001437 | 0.793727582057187 |
| frontal cortex - striatum | group | 1 | 24 | 0.545784389223331 | 6.61599363445805 | 1.97987272435351 | 0.172222846080703 | 0.258334269121054 |
| left frontal cortex - right frontal cortex | group | 1 | 24 | 0.310195070991261 | 10.5211433278535 | 0.707592461370745 | 0.40854761956015 | 0.40854761956015 |
| left striatum - right striatum | group | 1 | 24 | 1.26946452745239 | 6.96758534660439 | 4.37269830841832 | 0.0472817843627273 | 0.141845353088182 |
| frontal cortex - striatum | treatment | 1 | 24 | 0.152936046419061 | 6.61599363445805 | 0.554786675570634 | 0.463600351645728 | 0.851312232939882 |
| left frontal cortex - right frontal cortex | treatment | 1 | 24 | 0.0142345816970724 | 10.5211433278535 | 0.032470801897101 | 0.858511145516524 | 0.858511145516524 |
| left striatum - right striatum | treatment | 1 | 24 | 0.0975501825343306 | 6.96758534660439 | 0.336013735657347 | 0.567541488626588 | 0.851312232939882 |
| frontal cortex - striatum | time | 1 | 24 | 1.11297205234833 | 5.48057524345285 | 4.87381854455325 | 0.0370716329217311 | 0.0370716329217311 |
| left frontal cortex - right frontal cortex | time | 1 | 24 | 2.94198232282803 | 6.44868115294685 | 10.9491497677176 | 0.00294680879757391 | 0.00527564660304223 |
| left striatum - right striatum | time | 1 | 24 | 1.54487617031429 | 3.54003071720201 | 10.473645866228 | 0.00351709773536149 | 0.00527564660304223 |

DFn = Degrees of freedom in the numerator, DFd = degrees of freedom in the denominator, SSn = sum of squares in the numerator, SSd = sum of squares in the denominator, F= F-value, p = p-value, p.fdr = fdr-corrected p-value.

**Supplementary Table 4: Statistical results of mixed design ANOVA for structural connectivity measured with diffusion MRI.**

| variable | Effect | DFn | DFd | SSn | SSd | F | p | p.fdr |
| --- | --- | --- | --- | --- | --- | --- | --- | --- |
| frontal cortex - striatum | (Intercept) | 1 | 24 | 3.65951179627941 | 0.0142926521934519 | 6144.99547893176 | 1.94112967789936e-30 | 1.94112967789936e-30 |
| left striatum - right striatum | (Intercept) | 1 | 24 | 6.31994696496816 | 0.0133023455215998 | 11402.4047047152 | 1.1894685025282e-33 | 2.3789370050564e-33 |
| frontal cortex - striatum | group:treatment:time | 1 | 24 | 0.000336312478110478 | 0.0129153418501297 | 0.62495438125553 | 0.436954604441569 | 0.436954604441569 |
| left striatum - right striatum | group:treatment:time | 1 | 24 | 0.00035614309534129 | 0.0061646654510474 | 1.38652038071891 | 0.25053898525111 | 0.436954604441569 |
| frontal cortex - striatum | treatment:time | 1 | 24 | 0.000537789427088498 | 0.0129153418501297 | 0.999349951390896 | 0.327441003372531 | 0.327441003372531 |
| left striatum - right striatum | treatment:time | 1 | 24 | 0.00104943611376684 | 0.0061646654510474 | 4.08561777283873 | 0.0545380845201183 | 0.109076169040237 |
| frontal cortex - striatum | group:time | 1 | 24 | 0.000704836203430689 | 0.0129153418501297 | 1.30976547726196 | 0.263723080112885 | 0.52744616022577 |
| left striatum - right striatum | group:time | 1 | 24 | 1.56877862644519e-05 | 0.0061646654510474 | 0.0610749883082261 | 0.806906715249883 | 0.806906715249883 |
| frontal cortex - striatum | group:treatment | 1 | 24 | 0.000530508321038569 | 0.0142926521934519 | 0.890821348801789 | 0.354660132178269 | 0.709320264356539 |
| left striatum - right striatum | group:treatment | 1 | 24 | 6.19473151960586e-05 | 0.0133023455215998 | 0.111764918622157 | 0.741047576185823 | 0.741047576185823 |
| frontal cortex - striatum | group | 1 | 24 | 0.000105971144123936 | 0.0142926521934519 | 0.177945102458987 | 0.676899794550397 | 0.676899794550397 |
| left striatum - right striatum | group | 1 | 24 | 0.000758356572065314 | 0.0133023455215998 | 1.36822169443834 | 0.253603387077386 | 0.507206774154773 |
| frontal cortex - striatum | treatment | 1 | 24 | 0.00130174204156472 | 0.0142926521934519 | 2.18586505672239 | 0.152288307493397 | 0.304576614986794 |
| left striatum - right striatum | treatment | 1 | 24 | 0.000104820100759859 | 0.0133023455215998 | 0.18911570250162 | 0.667539025511131 | 0.667539025511131 |
| frontal cortex - striatum | time | 1 | 24 | 0.000871961118992247 | 0.0129153418501297 | 1.62032620573676 | 0.215239346915141 | 0.430478693830282 |
| left striatum - right striatum | time | 1 | 24 | 8.06385713952363e-05 | 0.0061646654510474 | 0.313938481958798 | 0.580466526177666 | 0.580466526177666 |

DFn = Degrees of freedom in the numerator, DFd = degrees of freedom in the denominator, SSn = sum of squares in the numerator, SSd = sum of squares in the denominator, F= F-value, p = p-value, p.fdr = fdr-corrected p-value.
